# Comparative effectiveness and certainty of exercise-based interventions for postpartum depressive symptoms: a systematic review and network meta-analysis of randomized controlled trials

**DOI:** 10.3389/fpsyt.2026.1889186

**Published:** 2026-08-03

**Authors:** Kun Chen, Jiahang Liu, Jian Zhang, Shijun Liu

**Affiliations:** 1 School of Sports Training, Tianjin University of Sport, Tianjin, China; 2 Office of Research and Graduate Education, Tianjin University of Sport, Tianjin, China; 3 School of Competitive Sports, Tianjin University of Sport, Tianjin, China

**Keywords:** exercise rehabilitation, exercise-based intervention, network meta-analysis, postpartum depression, randomized controlled trials

## Abstract

**Introduction:**

Postpartum depression is a common mental health condition after childbirth that can adversely affect maternal well-being, social functioning, and the mother–infant relationship. Although pharmacotherapy, psychotherapy, and exercise rehabilitation are available, current evidence is derived mainly from pairwise comparisons, leaving the relative efficacy of different exercise interventions and the certainty of comparative evidence unclear. This study therefore conducted a systematic review and network meta-analysis to compare the effects of different exercise rehabilitation interventions on postpartum depression and to evaluate the certainty of the evidence for clinical decision-making and exercise prescription.

**Methods:**

This systematic review and network meta-analysis was conducted according to a prespecified protocol. PubMed, Embase, the Cochrane Library, and Web of Science were systematically searched from inception to March 1, 2026. Randomized controlled trials involving postpartum women were eligible for inclusion. Interventions were classified as active control (AC), exercise combined with other interventions (E+CI), lifestyle physical activity promotion (LPA), mind–body exercise (MBE), passive control (PC), pelvic floor muscle training (PFMT), resistance training (RT), and structured aerobic exercise (SAE). The primary outcome was postpartum depression score treated as a continuous variable. Two reviewers independently screened studies, extracted data, and assessed risk of bias. A frequentist random-effects network meta-analysis was conducted to synthesize direct and indirect evidence, with results expressed as standardized mean differences (SMDs) and 95% confidence intervals (CIs). Treatments were ranked using the surface under the cumulative ranking curve (SUCRA), the probability of being the best treatment, and mean rank. The certainty of evidence was evaluated using Confidence in Network Meta-Analysis (CINeMA) and Grading of Recommendations Assessment, Development and Evaluation (GRADE).

**Results:**

A total of 23 randomized controlled trials involving 2,752 postpartum women and 8 intervention/control nodes were included. Most studies were judged as having some concerns, and a few were rated as high risk of bias. No statistically significant global inconsistency was identified. For the primary outcome, SAE significantly reduced postpartum depression scores compared with both passive control and active control, showing the clearest statistically supported comparative effects. MBE showed favorable point estimates and ranking-based performance; however, its key comparisons with passive and active control did not reach statistical significance. Subgroup analyses suggested that MBE may be more favorable among postpartum women with baseline depression, whereas SAE may be more favorable among women without baseline depression who were considered high-risk or included in preventive samples. Sensitivity analyses suggested that SAE was relatively robust, whereas MBE was more sensitive to the influence of individual studies. Overall, the certainty of evidence was low to very low.

**Conclusion:**

Among postpartum women, SAE showed the most consistent statistically significant advantages in key comparisons and demonstrated greater robustness in sensitivity analyses. MBE also appeared promising based on favorable point estimates and ranking-based results, but its main comparisons with passive and active control were not statistically significant and should not be interpreted as definitive evidence of superiority. Therefore, current evidence suggests that SAE has the clearest comparative support, while MBE remains a potentially beneficial intervention that requires further confirmation. However, the available evidence is limited by risk of bias, small sample sizes, imprecision, and low overall certainty. Further high-quality randomized controlled trials with larger samples, head-to-head comparisons, and standardized outcome reporting are required to confirm the relative efficacy of different exercise rehabilitation interventions.

**Systematic review:**

https://www.crd.york.ac.uk/PROSPERO/, identfier CRD420261352226.

## 1 Background

Postpartum depression (PPD) is a depressive disorder that occurs after childbirth and is typically characterized by persistent low mood, diminished interest, anxiety, self-blame, fatigue, and disturbances in sleep and appetite. It may also adversely affect mother–infant interaction, family functioning, and social functioning (1, 2). Perinatal depression may occur during pregnancy or within the first year postpartum, and PPD is one of its most clinically prominent forms. Epidemiological evidence suggests that PPD is common worldwide, with prevalence estimates generally ranging from 10% to 20% and an overall prevalence of approximately 17% (3, 4), although considerable regional and national variation has been reported. In addition to compromising maternal mental health and caregiving capacity, PPD has been linked to poorer infant care, impaired mother–infant bonding, and unfavorable long-term developmental outcomes in offspring, thereby imposing a substantial burden on healthcare systems and society (1–5).

Current standard treatments for PPD primarily include psychotherapy and pharmacotherapy. Cognitive behavioral therapy (CBT), interpersonal psychotherapy (IPT), and antidepressant medications are widely used in the management of perinatal and postpartum depression (1–5). However, despite their established efficacy, the uptake and sustained benefit of these treatments are often limited by multiple real-world barriers. Women may delay or decline treatment because of concerns about medication safety during breastfeeding, fear of adverse effects, and stigma associated with psychiatric care. Psychotherapy is further constrained by shortages of trained professionals, limited accessibility, relatively long treatment courses, and high cost. Consequently, PPD remains underrecognized, undertreated, and inadequately managed in routine clinical practice (1, 5).

Exercise rehabilitation has attracted increasing attention in the management of PPD as a low-cost, scalable, and non-pharmacological approach that offers both physical and psychological benefits (6–10). Exercise may improve depressive symptoms through multiple pathways, including behavioral activation, better sleep, reduced fatigue, enhanced self-efficacy and social support, and modulation of inflammatory responses, neuroplasticity, and autonomic nervous system function. A growing body of evidence suggests that regular physical activity and exercise rehabilitation may be important non-pharmacological strategies for promoting mental health during the perinatal and postpartum periods (6–8). However, existing research has focused mainly on whether exercise is beneficial, with less consensus on which type of exercise is most effective and what frequency and duration are most appropriate for postpartum depression rehabilitation (6–8).

Previous systematic reviews and meta-analyses have generally supported the beneficial effects of exercise on postpartum depressive symptoms, but the relative efficacy of different exercise modalities remains uncertain (9, 10). Most existing studies have focused on pairwise comparisons between a single exercise modality and usual care and thus have rarely addressed the clinically relevant question of which type of exercise is most effective. Some have also placed undue emphasis on the SUCRA rankings while giving insufficient attention to evidence certainty, the precision of effect estimates, and the robustness of the findings. In addition, therapeutic and preventive samples have often been pooled, and inconsistencies in exercise classification, control definitions, and outcome measures further limit the clinical interpretability of the available evidence (9, 10).

Network meta-analysis allows the integration of direct and indirect evidence within a single analytical framework, enabling the relative comparison of multiple exercise rehabilitation interventions (10). Accordingly, this study conducted a systematic review and network meta-analysis to compare the effects of different exercise rehabilitation modalities on postpartum depression outcomes. By incorporating risk-of-bias assessment, sensitivity analyses, subgroup analyses, and certainty-of-evidence evaluation, the study sought to provide clinically relevant evidence to guide exercise rehabilitation strategies for postpartum depression.

## 2 Materials and methods

### 2.1 Data sources and search strategy

This study was conducted as a network meta-analysis (NMA) in accordance with the Preferred Reporting Items for Systematic Reviews and Meta-Analyses extension statement for network meta-analyses (PRISMA-NMA) (11). To enhance transparency, methodological rigor, and reproducibility, the protocol was prospectively registered in the International Prospective Register of Systematic Reviews (PROSPERO; CRD420261352226).

The literature search was independently performed by two reviewers using a structured search strategy, and the completeness and reproducibility of the search syntax were verified by a third reviewer. PubMed, Embase, the Cochrane Library, and Web of Science Core Collection were systematically searched from inception to March 1, 2026. No restrictions were imposed on publication language, country, or region. To minimize the risk of missing relevant studies, supplementary searches were conducted in ClinicalTrials.gov, the World Health Organization International Clinical Trials Registry Platform (WHO ICTRP), and PROSPERO to identify completed but unpublished studies, ongoing trials, and potentially duplicate registrations.

The search strategy was built around four core concepts: condition, population, intervention, and study design. Condition-related terms included “postpartum depression,” “postnatal depression,” “perinatal depression,” and “depressive symptoms.” Intervention-related terms included “exercise,” “physical activity,” “aerobic exercise,” “resistance training,” “pelvic floor muscle training,” “mind–body exercise,” “yoga,” “tai chi,” “breathing training,” “exercise support,” and “health promotion.” These were combined with study design terms such as “randomized controlled trial,” “randomised controlled trial,” and “RCT.” Search strings were adapted to the indexing terms and syntax requirements of each database. The complete database-specific search strategies, including the exact search strings, search dates, field restrictions, controlled vocabulary terms such as MeSH and Emtree, and the use of randomized controlled trial (RCT) filters, are presented in **Supplementary Table S1**. For ClinicalTrials.gov, WHO ICTRP, and PROSPERO, simplified condition- and intervention-related search strategies were used to identify completed, ongoing, unpublished, or registered studies.

### 2.2 Eligibility criteria

#### 2.2.1 Included studies

1) Participants were postpartum women recruited or enrolled within the first 12 months after childbirth. This eligibility window was selected because postpartum/perinatal depression is commonly defined and managed within the first postpartum year in clinical and epidemiological literature. For trials with interventions or follow-up assessments extending beyond 12 months postpartum, eligibility was determined according to the timing of recruitment or intervention initiation rather than the timing of the final outcome assessment. Eligibility was not restricted by baseline depressive status. Studies were eligible if they included women with clinically diagnosed postpartum depression or elevated postpartum depressive symptoms identified by diagnostic criteria or screening instruments, and women who did not meet diagnostic or screening thresholds for depression but were considered at high risk for postpartum depression or targeted for preventive intervention. Depressive status was determined according to the diagnostic criteria or scale cut-off values reported in the original studies, including but not limited to the Edinburgh Postnatal Depression Scale (EPDS), Hamilton Depression Rating Scale (HAM-D/HDRS), Center for Epidemiologic Studies Depression Scale (CES-D), Patient Health Questionnaire-8/9 (PHQ-8/PHQ-9), Structured Clinical Interview for DSM Perinatal Version (SCID-PN), and ICD-10. 2) The intervention group received an intervention explicitly related to exercise, physical activity, or exercise rehabilitation. To improve the clinical coherence and transitivity of intervention nodes in the network meta-analysis, exercise interventions were prespecified and classified into six categories based on their core components, exercise modality, intervention objective, and co-intervention characteristics: lifestyle physical activity promotion (LPA), structured aerobic exercise (SAE), mind–body exercise (MBE), resistance training (RT), pelvic floor muscle training (PFMT), and exercise combined with other interventions (E+CI). LPA was defined as flexible activity-promotion interventions primarily aimed at increasing daily physical activity. SAE was defined as structured aerobic exercise delivered according to a prespecified exercise prescription. MBE was defined as exercise modalities combining physical activity with breathing regulation and/or psychological regulation. RT was defined as interventions centered on strength or resistance training. PFMT was defined as exercise interventions primarily focused on pelvic floor muscle training or pelvic floor rehabilitation. E+CI was defined as multicomponent interventions in which exercise was combined with diet, psychological support, health promotion, or other non-exercise components, such that the independent effect of exercise could not be isolated. Accordingly, E+CI was retained as a distinct multicomponent node rather than merged with other exercise-only nodes. Its comparative effects and ranking results were interpreted as the effects of a combined intervention package, rather than the independent effect of exercise alone. When an intervention met the criteria for more than one node, it was classified according to its primary objective and core component. Classification was performed independently by two reviewers, and disagreements were resolved through discussion or consultation with a third reviewer. To further reduce subjectivity in node assignment, the classification followed prespecified decision rules based on the primary intervention objective, core exercise component, delivery format, and whether non-exercise components were essential to the intervention effect. Delivery format alone was not used to define a node. Therefore, yoga, laughter yoga, and virtual reality (VR)-enhanced mindfulness plus yoga were classified as MBE because they all combined physical movement with breathing, attentional, emotional, or mindfulness-related regulation components. PFMT was retained as a separate exercise-based rehabilitation node because it involved prescribed and repeated pelvic floor muscle contractions and is commonly delivered as postpartum physical rehabilitation; however, its potential effect on depressive symptoms was interpreted cautiously because it was not primarily designed as a depression-specific psychological intervention. Two reviewers independently re-evaluated the intervention-node assignment of all included studies according to the revised prespecified classification criteria. The initial agreement was 22/23 studies (95.7%). The only discrepancy concerned the classification of a multicomponent physical activity promotion intervention as LPA or E+CI. This discrepancy was resolved through discussion based on the primary intervention objective and core therapeutic component. 3) Control conditions were categorized as passive control (PC) and active control (AC). The distinction between PC and AC was based on whether the comparator involved structured trial-delivered contact or support beyond routine postpartum management. PC included wait-list control, no intervention, and usual or standard postpartum care without additional structured education, attention, social support, health promotion, or exercise components. AC included health education, attention control, social support, wellness/support contact, general health promotion, education-only comparators, and non-primary exercise comparators involving structured contact or activity. Accordingly, usual care combined with structured health education or planned supportive contact was classified as AC, whereas routine postpartum management without additional structured content was classified as PC. Detailed definitions and classification criteria are presented in **Supplementary Table S2**, and study-level control-condition judgments are provided in **Supplementary Table S3**. (4) Eligible study designs were randomized controlled trials, including individually randomized trials, cluster-randomized trials, and multi-arm randomized controlled trials. For multi-arm trials, data from each arm were extracted, and within-study correlations were accounted for in the network meta-analysis to avoid double-counting shared control groups. 5) Studies were required to report at least one continuous postpartum depression-related outcome and to provide the mean, standard deviation, and sample size needed for meta-analysis, or sufficient information to derive these values.

#### 2.2.2 Exclusion criteria

Studies were excluded if they met any of the following criteria: 1) the study population was not composed of postpartum women; 2) the report was a duplicate publication or included a duplicate or overlapping study population; 3) the study used a non-randomized, single-arm, observational, case-report, protocol, conference-abstract, or animal-study design; 4) the intervention was not clearly related to exercise or physical activity; 5) the control condition was inappropriate or did not meet the prespecified eligibility criteria; 6) no eligible postpartum depression outcome was reported; or 7) quantitative data were insufficient to derive the mean, standard deviation, and sample size required for meta-analysis.

### 2.3 Data extraction and quality assessment

Two reviewers independently extracted data using a prespecified data extraction form. The extracted variables included first author, publication year, country or region, sample size, mean age, baseline depressive status, intervention details, control conditions, intervention duration, intervention frequency, and depression outcome data. For continuous outcomes, post-intervention means and standard deviations were preferentially extracted.

Methodological quality was assessed using the Cochrane Risk of Bias 2.0 tool (RoB 2.0) (12), which evaluates bias across five domains: the randomization process, deviations from intended interventions, missing outcome data, measurement of the outcome, and selection of the reported result. Each domain, as well as the overall risk of bias, was rated as low risk, some concerns, or high risk. Quality assessment was conducted independently by two reviewers, and disagreements were resolved through discussion.

### 2.4 Statistical analysis

All network meta-analyses were performed using Stata/MP 18.0. Because the scales used to assess postpartum depression were not uniform across the included studies, standardized mean differences (SMDs) with 95% confidence intervals (CIs) were used as the primary effect measure. When the same outcome scale and unit were applied, mean differences (MDs) were additionally reported for descriptive comparison. For multi-arm trials, the network was specified in augmented format, and within-study correlations were retained during model estimation to avoid underestimation of standard errors due to repeated use of a shared control group. The primary analysis was conducted under the consistency assumption using a random-effects model, with the between-study variance (τ^2^) estimated by restricted maximum likelihood (REML).

When closed loops were present in the network, global inconsistency was assessed to examine overall agreement between direct and indirect evidence, and local inconsistency was evaluated using the node-splitting approach. A p-value < 0.05 was considered indicative of potential inconsistency. In addition, loop-specific inconsistency factors (IFs) were calculated to assess agreement within each closed loop; if the 95% CI of the IF included 0, no important inconsistency was considered present. Network plots were generated to illustrate the geometry of the intervention network, with node size proportional to the total sample size for each intervention and edge thickness proportional to the number of studies contributing direct comparisons. Treatment ranking was evaluated using the surface under the cumulative ranking curve (SUCRA), the probability of being the best treatment (PrBest), and mean rank. Publication bias and small-study effects were assessed using comparison-adjusted funnel plots when more than 10 studies were available. As a quantitative complement to visual inspection, Egger’s regression test was performed to statistically assess funnel plot asymmetry, with a p-value < 0.05 indicating potential publication bias or small-study effects. Robustness of the findings was further examined through leave-one-out sensitivity analyses and univariable network meta-regression, with prespecified covariates including mean age, country or region, intervention duration, and intervention frequency.

### 2.5 Certainty of evidence assessment

The certainty of evidence for the network meta-analysis was assessed using the GRADE framework in conjunction with the Confidence in Network Meta-Analysis (CINeMA) approach (13, 14). Evidence from randomized controlled trials was initially considered high certainty and was subsequently evaluated across six domains: within-study bias, indirectness, imprecision, heterogeneity, inconsistency, and across-study bias. The overall certainty of evidence was ultimately rated as high, moderate, low, or very low.

## 3 Results

### 3.1 Study selection and characteristics

**Figure 1:**
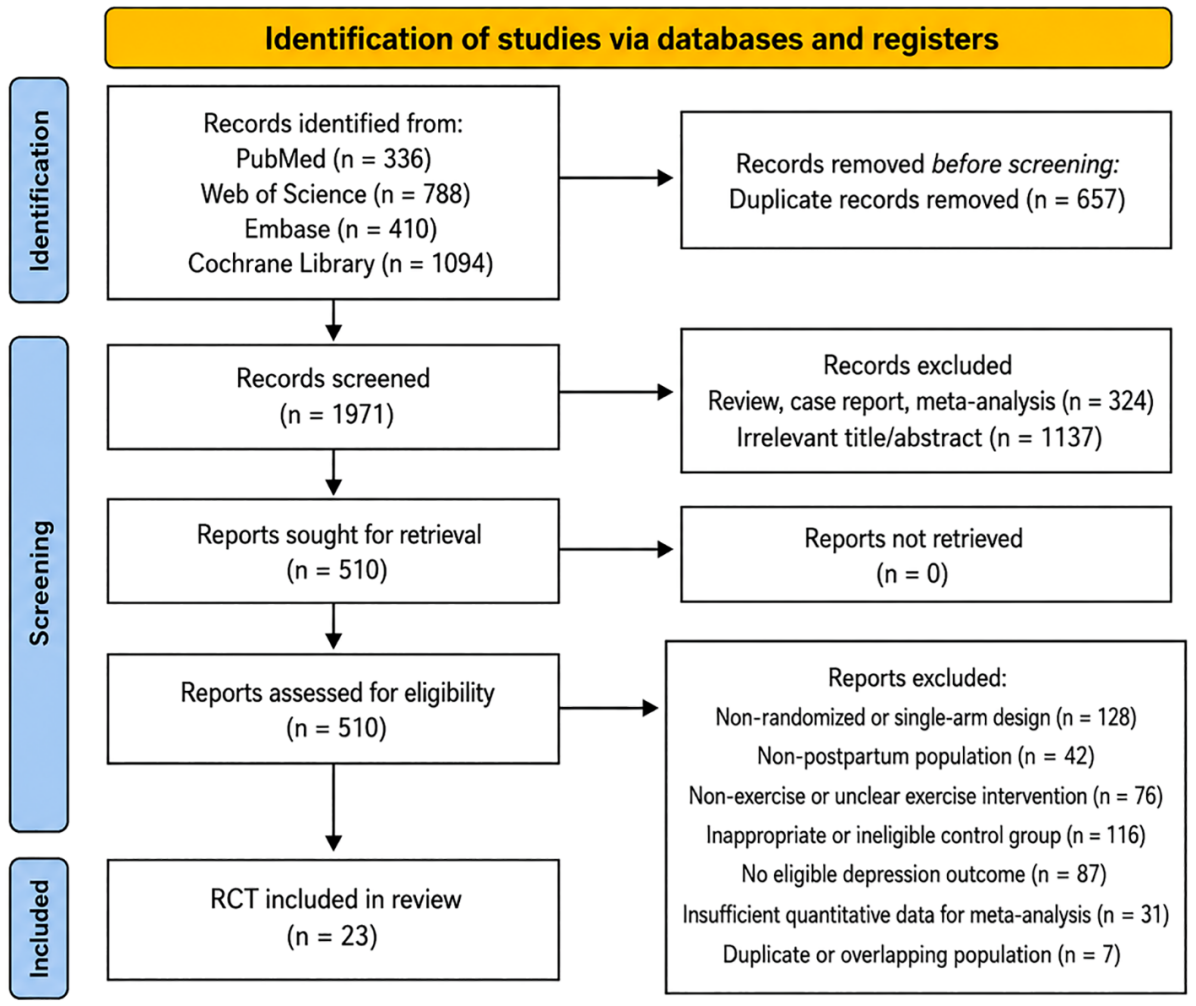
Literature search and study selection flow diagram. RCT, randomized controlled trial.

Following systematic retrieval and stepwise screening, 23 RCTs were included in the final analysis (15–37). The study selection process is presented in **Figure 1**. Among the 510 full-text reports assessed for eligibility, 487 were excluded because of non-randomized or single-arm design, non-postpartum populations, non-exercise or unclear exercise interventions, inappropriate or ineligible control conditions, absence of an eligible depression outcome, insufficient quantitative data for meta-analysis, or duplicate or overlapping populations. Overall, the included studies involved 2,752 postpartum women.

The evidence network comprised eight intervention/control nodes—AC, E+CI, LPA, MBE, PC, PFMT, RT, and SAE—yielding 10 direct comparisons. The included studies were published between 2001 and 2025 and were conducted in the United States, Australia, the United Kingdom, Taiwan, Turkey, Canada, Japan, India, mainland China, and multicenter settings spanning the United Kingdom and New Zealand. The mean age of participants was approximately 29.8 years. Intervention duration ranged from 4 to 52 weeks, with intervention frequency ranging from 1 to 7 sessions per week. Detailed study characteristics are summarized in **Table 1**, detailed interventions and outcome measures are presented in **Table 2**, and study-level control-condition judgments are provided in **Supplementary Table S3**.

**Table 1:** Baseline characteristics of the included studies.

First author and year	Country/region	Postpartum timing	Intervention group (n)	Control group (n)	Age (years)	Baseline depressive status	Intervention duration (weeks)	Frequency (sessions/week)
Keller, 2014 (15)	United States	6 weeks to 6 months postpartum	71	68	28.3	No baseline depression, but high-risk or preventive sample	52	3
Forsyth, 2017 (16)	United Kingdom	Enrolled after routine 6-week postpartum screening	11	11	26.0	Baseline depression/depressive symptoms above threshold	12	2
Da Costa, 2009 (17)	Canada	11–12 weeks postpartum	46	42	33.5	Baseline depression/depressive symptoms above threshold	12	3
Daley, 2008 (18)	United Kingdom	Within 12 months after childbirth	16	15	30.5	Baseline depression/depressive symptoms above threshold	12	1
Glazener, 2001	United Kingdom/New Zealand	3 months postpartum	238	219	29.5	No baseline depression, but high-risk or preventive sample	36	7
Armstrong, 2004 (20)	Australia	Within 12 months postpartum	9	10	30.0	Baseline depression/depressive symptoms above threshold	12	3
Heh, 2008 (21)	Taiwan, China	Screened at 4 weeks postpartum; intervention started at 6 weeks	33	30	27.5	Baseline depression/depressive symptoms above threshold	12	3
Buttner, 2015 (22)	United States	Approximately 4.6 months postpartum	28	29	31.1	Baseline depression/depressive symptoms above threshold	8	2
Teychenne, 2021 (23)	Australia	3–9 months postpartum	31	25	33.3	Baseline depression or high-risk sample	12	3
Daley, 2015 (24)	United Kingdom	Within the first 6 months postpartum	47	47	30.5	Baseline depression/depressive symptoms above threshold	24	1
Lewis, 2014 (25)	United States	Early postpartum/high-risk preventive sample	61	61	31.5	No baseline depression, but high-risk or preventive sample	24	5
Lewis, 2021 (26)	United States	Mean postpartum time approximately 4 weeks	150	150	30.7	No baseline depression, but high-risk or preventive sample	24	5
Özkan, 2020 (27)	Turkey	Postpartum women	34	31	28.9	Baseline depression/depressive symptoms above threshold	4	4
Norman, 2010 (28)	Australia	Postpartum women	62	73	29.7	No baseline depression, but high-risk or preventive sample	8	1
Surkan, 2012 (29)	United States	Low-income multiethnic postpartum women	200	203	26.5	No baseline depression, but high-risk or preventive sample	52	5
Yang, 2018 (30)	Taiwan, China	6 weeks postpartum	70	70	31.9	No baseline depression, but high-risk or preventive sample	12	3
LeCheminant, 2014 (31)	United States	Postpartum women	21	23	26.4	No baseline depression, but high-risk or preventive sample	18	2
Armstrong, 2003 (32)	Australia	Postpartum women	10	10	25.5	Baseline depression/depressive symptoms above threshold	12	3
Haruna, 2013 (33)	Japan	3 months postpartum	48	47	33.8	No baseline depression, but high-risk or preventive sample	4	4
Thiruppathi, 2014 (34)	India	Postpartum primiparous women	20	21	25.7	No baseline depression, but high-risk or preventive sample	4	3
Ko, 2008 (35)	Taiwan, China	1 month postpartum	31	30	34.3	No baseline depression, but high-risk or preventive sample	3	3
Koca, 2025 (36)	Turkey	Postpartum women	60	44	29.75	Baseline depression/depressive symptoms above threshold	6	1
Liu, 2025 (37)	China	Postpartum women	29	28	30.5	No baseline depression, but high-risk or preventive sample	8	3

**Table 2:** Detailed interventions and outcome measures of the included studies.

First author and year	Detailed intervention in the intervention group	Detailed intervention in the control group	Depression outcome measure
Keller, 2014 (15)	Social support-facilitated moderate-intensity physical activity program centered on walking/physical activity promotion, combined with weight management and behavioral support.	Attention control/routine health information without equivalent exercise promotion.	Depression score
Forsyth, 2017 (16)	Pram-walking, group exercise, and self-initiated home exercise as an adjunctive treatment for postpartum depression.	Usual care.	EPDS
Da Costa, 2009 (17)	12-week individualized home-based exercise program supervised by professionals.	Usual care.	HAM-D
Daley, 2008 (18)	2 one-to-one exercise consultations plus telephone support to encourage gradual establishment of regular moderate-intensity activity, mainly including walking and pram-pushing.	Usual care.	EPDS
Glazener, 2001	Nurse assessment plus pelvic floor muscle training, with additional bladder training when necessary.	Standard management.	HAD depression
Armstrong, 2004 (20)	Pram-walking program.	Social support group involving non-structured supportive activities.	EPDS
Heh, 2008 (21)	Exercise support program: 1 h per week in hospital plus home exercise twice weekly.	Standard care.	EPDS
Buttner, 2015 (22)	Yoga intervention, 16 sessions over 8 weeks.	Wait-list control.	HDRS
Teychenne, 2021 (23)	Home-based multicomponent physical activity promotion program including free equipment, smartphone app, online forum, and logbook.	Usual routine.	EPDS
Daley, 2015 (24)	Facilitated exercise intervention involving two face-to-face consultations and two telephone support sessions.	Usual care only.	EPDS
Lewis, 2014 (25)	Telephone-delivered home-based physical activity intervention lasting 6 months.	Wellness/support contact control discussing stress, sleep, nutrition, and related topics, but not focused on exercise promotion.	EPDS
Lewis, 2021 (26)	Telephone-based exercise intervention.	Wellness/support intervention plus usual care; active control arm in a three-arm trial.	EPDS
Özkan, 2020 (27)	4-week structured exercise program.	Standard care.	EPDS
Norman, 2010 (28)	Mother and Baby program: exercise plus health care education.	Education only.	EPDS
Surkan, 2012 (29)	Just for You (JFY) health promotion program including home visits and motivational telephone counseling to promote diet, physical activity, self-efficacy, and social support.	Usual Special Supplemental Nutrition Program for Women, Infants, and Children (WIC) care.	CES-D
Yang, 2018 (30)	Home-based aerobic gymnastic exercise at least 3 times per week, 15 min per session.	Routine care/control.	EPDS
LeCheminant, 2014 (31)	Resistance training twice weekly for 18 weeks.	Flexibility training (active control).	CES-D
Armstrong, 2003 (32)	Multicomponent program combining exercise and social support.	Non-intervention control.	EPDS
Haruna, 2013 (33)	Ball-exercise classes once weekly for 90 min over 4 weeks.	Control/usual care.	EPDS
Thiruppathi, 2014 (34)	Structured physical activity plus healthcare education.	Healthcare education only.	EPDS
Ko, 2008 (35)	Low-intensity postpartum exercise program in a postpartum care center, with at least 6 completed sessions.	Traditional postpartum care without a physical activity component.	CES-D
Koca, 2025 (36)	Laughter yoga, delivered in 6 sessions.	Control group without laughter yoga.	EPDS
Liu, 2025 (37)	VR-enhanced mindfulness plus yoga, 3 times per week, 60 min per session.	Traditional in-person sessions/active control; part of a three-arm trial.	EPDS

EPDS, Edinburgh Postnatal Depression Scale; HAM-D/HDRS, Hamilton Depression Rating Scale; CES-D, Center for Epidemiologic Studies Depression Scale.

### 3.2 Risk of bias assessment

**Figure 2:**
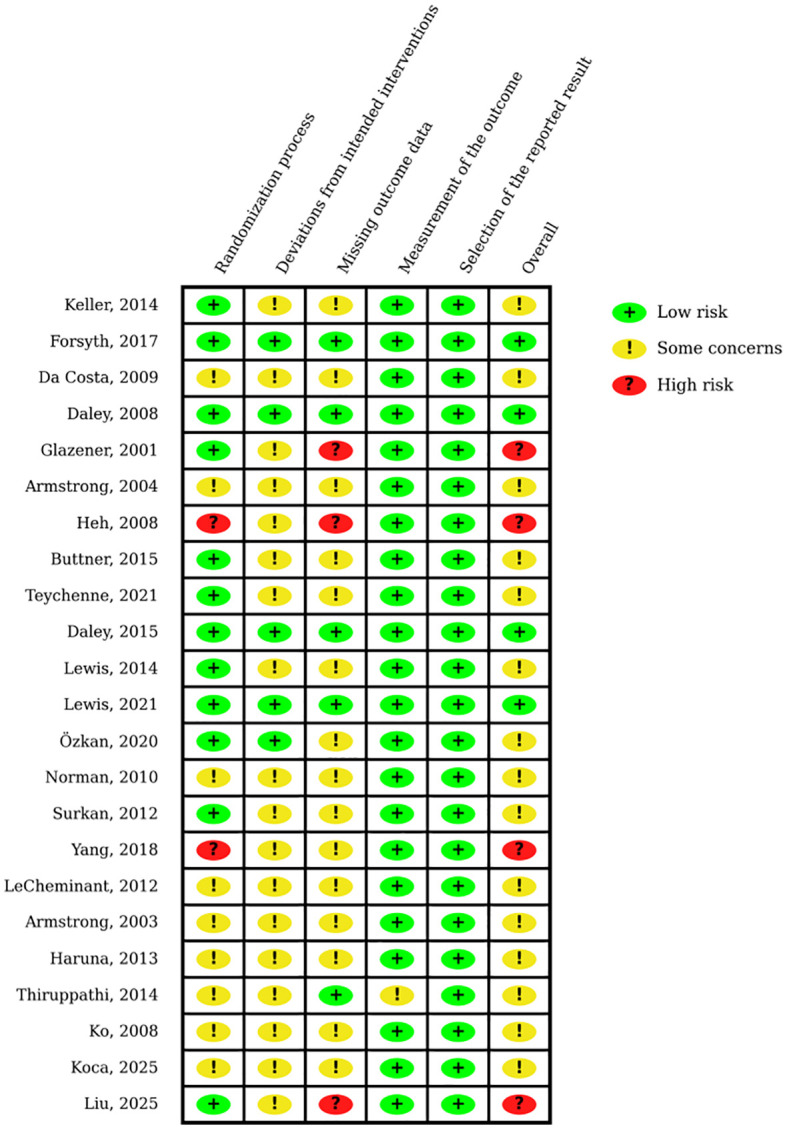
RoB 2.0 quality assessment of included studies. RoB, Cochrane Risk of Bias.

Risk of bias in the 23 included randomized controlled trials was assessed using the RoB 2 tool. Overall, four studies were judged to be at low risk of bias, 15 were judged as having some concerns, and 4 were rated as high risk, indicating moderate methodological limitations in the included evidence (**Figure 2**).

For the randomization process, 12 studies were assessed as low risk of bias, 9 as having some concerns, and 2 as high risk. For deviations from intended interventions, 5 studies were rated as low risk and 18 as having some concerns. For missing outcome data, 5 studies were classified as low risk, 15 as having some concerns, and 3 as high risk. For measurement of the outcome, 22 studies were rated as low risk and 1 as having some concerns. For the selection of the reported result, all 23 studies were judged to be at low risk. Overall, the methodological quality of the included studies was moderate, with the main concerns related to the difficulty of implementing full blinding in exercise interventions and the presence of missing outcome data in some studies.

### 3.3 Consistency and heterogeneity

**Figure 3:**
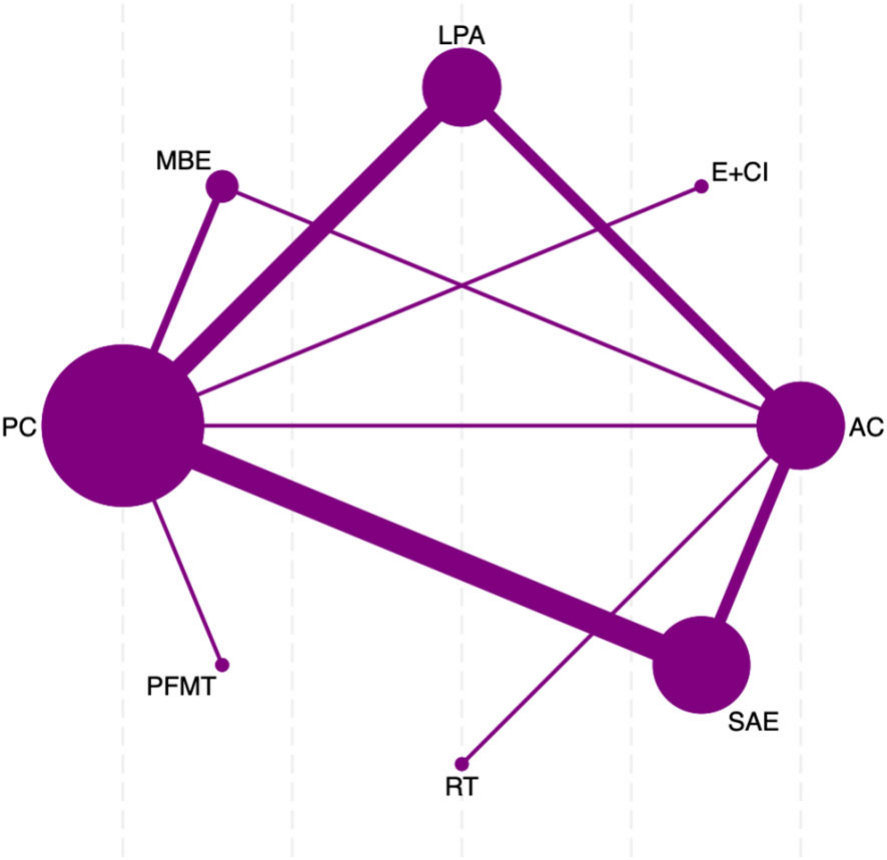
Network plot of exercise interventions for postpartum depression. Node size is proportional to the total sample size for each intervention, and line thickness is proportional to the number of direct comparisons. AC, active control; E+CI, exercise combined with other interventions; LPA, lifestyle physical activity promotion; MBE, mind-body exercise; PC, passive control; PFMT, pelvic floor muscle training; RT, resistance training; SAE, structured aerobic exercise.

The network for postpartum depression scores comprised eight nodes and 10 direct comparisons. Because several closed loops were present, including AC–LPA–PC, AC–SAE–PC, and AC–MBE–PC, consistency was formally assessed. The global inconsistency test was not statistically significant (χ^2^ = 3.61, p = 0.4619), indicating no evidence of inconsistency between direct and indirect evidence within the network. The network structure is shown in **Figure 3**. Accordingly, subsequent analyses were performed under the consistency assumption using a random-effects network meta-analysis model.

Based on the baseline characteristics summarized in **Table 1**, the included studies varied to some extent in mean age, baseline depressive status, postpartum timing, and intervention duration. Nonetheless, the overall level of clinical comparability was considered acceptable, supporting the plausibility of the transitivity assumption. As some heterogeneity remained across studies with respect to exercise modality, frequency, and duration, a random-effects model was used to account for between-study variability.

Heterogeneity analysis indicated that the between-study standard deviation (τ) for the depression outcome was 0.852, suggesting the presence of moderate heterogeneity across studies. The overall I^2^ was 53%, which also indicates a moderate level of heterogeneity. Therefore, the pooled effect estimates for depressive outcomes should be interpreted with caution under a random-effects model.

### 3.4 Depression score

For the primary outcome of postpartum depression scores, data from 23 studies were available across eight intervention/control conditions in postpartum women. Because depression rating scales were not uniform across studies, effect estimates were expressed as SMDs.

**Figure 4:**
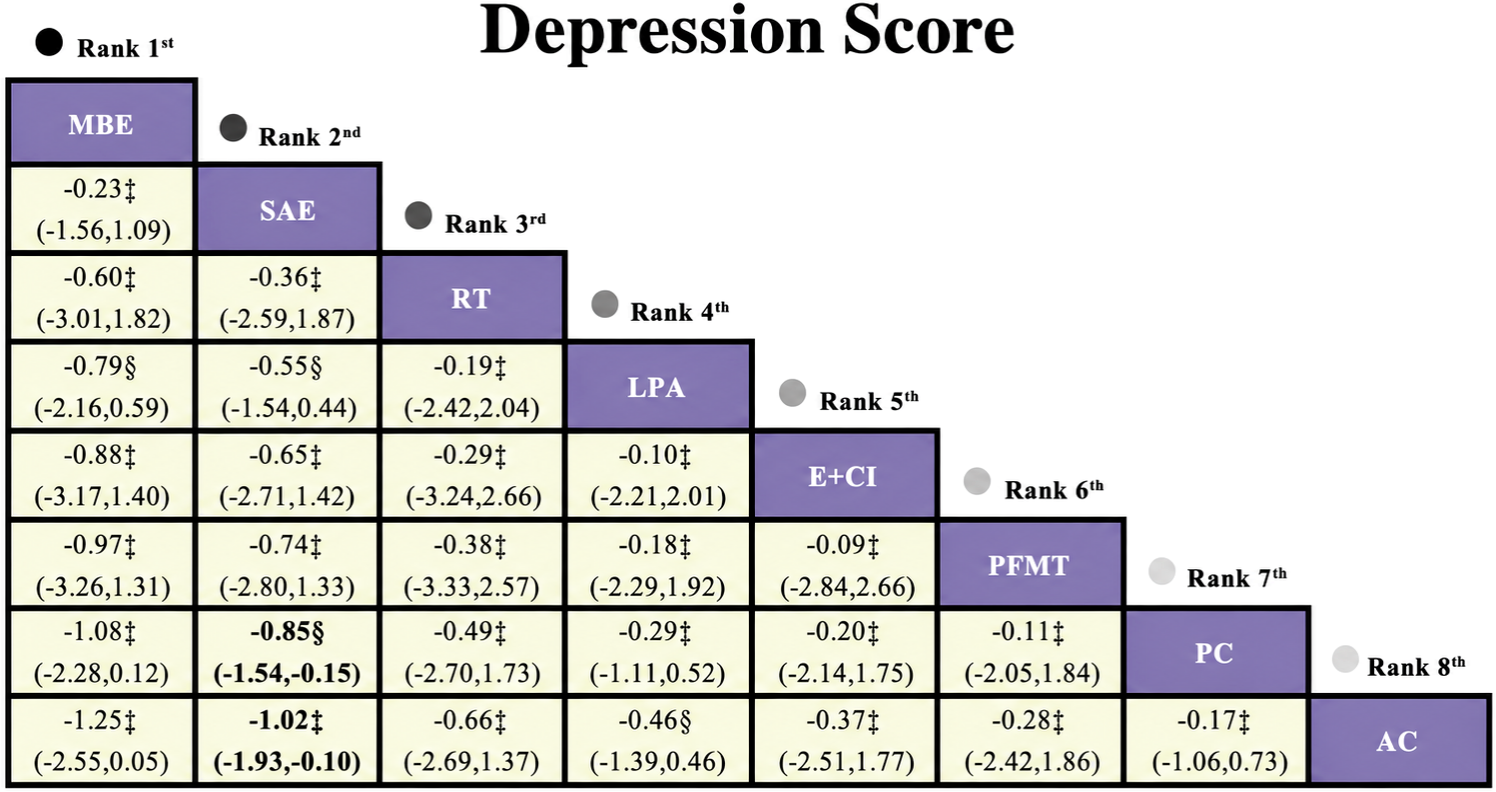
League table of network meta-analysis comparing exercise interventions for depression score. Note: SMDs and 95% CIs are presented in the lower-left triangle. Bold values indicate statistically significant differences. The symbols indicate the certainty of evidence: ‡ = low certainty and § = very low certainty. AC, active control; E+CI, exercise combined with other interventions; LPA, lifestyle physical activity promotion; MBE, mind-body exercise; PC, passive control; PFMT, pelvic floor muscle training; RT, resistance training; SAE, structured aerobic exercise; CI, confidence interval; SMD, standardized mean difference.

**Figure 5:**
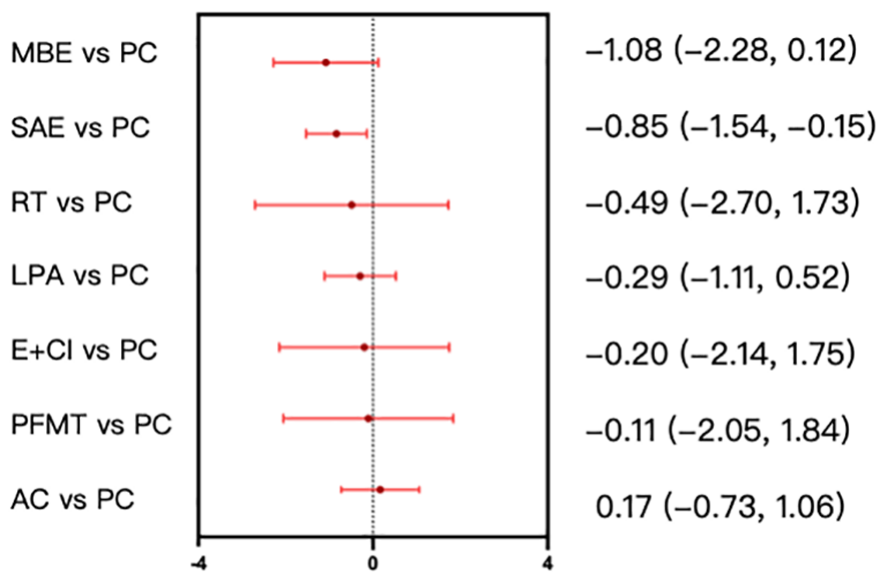
Forest-style plot of network estimates for depression score versus passive control. Note: Effect estimates are presented as standardized mean differences (SMDs) with 95% confidence intervals. Negative values favor the intervention, whereas positive values favor passive control (PC).

Network meta-analysis showed that SAE had clear advantages in the key comparisons. Compared with PC, SAE significantly reduced depression scores (SMD = −0.85, 95% CI: −1.54 to −0.15). SAE also showed a statistically significant benefit over AC (SMD = −1.02, 95% CI: −1.93 to −0.10). MBE showed a similarly favorable direction of effect in the point estimates; however, its 95% confidence intervals crossed the line of no effect in comparisons with both PC and AC, and the results did not reach statistical significance. The remaining interventions also tended to favor exercise-based interventions, but their 95% confidence intervals generally crossed the null line, indicating no statistically significant differences (**Figure 4**). To provide a more intuitive visual summary of the main comparisons, a forest-style plot of all interventions versus PC was added and is presented in **Figure 5**.

With respect to treatment ranking, SUCRA values placed MBE first (81.0), followed by SAE (76.2), RT (54.5), LPA (48.7), E+CI (45.1), PFMT (41.5), PC (30.5), and AC (22.5). The PrBest analysis showed that MBE had the highest value (37.6%), followed by RT (21.7%) and SAE (15.6%). Mean rank results were broadly consistent with these findings, with MBE showing the lowest mean rank (2.3), followed by SAE (2.7) and RT (4.2). Taken together, MBE was the highest-ranked intervention according to the SUCRA, followed by SAE. However, the ranking of MBE should be interpreted cautiously because its key comparisons with passive and active control did not reach statistical significance, and the certainty of evidence was limited. In contrast, SAE showed statistically significant advantages in key comparisons and greater robustness in sensitivity analyses.

### 3.5 Subgroup analysis

Among postpartum women with baseline depression or depressive symptoms above the prespecified threshold, 11 studies involving 619 participants were included, yielding a five-node network comprising AC, LPA, MBE, PC, and SAE. Ranking results indicated that MBE had the highest SUCRA value in this subgroup (89.5), with a PrBest value of 71.7% and a mean rank of 1.4; however, this ranking should not be interpreted as definitive evidence of superiority. SAE had a SUCRA value of 68.7 and a mean rank of 2.3. In pairwise comparisons, MBE showed a statistically significant advantage over LPA (SMD = −1.60, 95% CI: −2.95 to −0.26). Most of the remaining comparisons also favored MBE or SAE in terms of point estimates, although their 95% confidence intervals crossed the line of no effect and did not reach statistical significance. Taken together, these findings suggest that MBE and SAE may offer relative advantages among postpartum women with baseline depression, with MBE showing a relatively favorable ranking profile in this subgroup, although the statistical evidence remained limited for several comparisons (**Supplementary Figure S2**).

Among postpartum women without baseline depression but considered high-risk or included in preventive samples, 12 studies involving 2,133 participants were included, yielding an eight-node network. Ranking analysis showed that SAE had the highest SUCRA value (76.9), followed by E+CI (62.0) and PFMT (58.8). In the PrBest analysis, E+CI, SAE, and PFMT had the highest probabilities at 25.7%, 22.9%, and 22.4%, respectively. The league table further showed that most pairwise comparisons in this subgroup did not reach statistical significance. Although SAE, E+CI, and PFMT generally showed more favorable point estimates, their 95% confidence intervals mostly crossed the line of no effect. Overall, these findings suggest that SAE, E+CI, and PFMT may offer relative advantages in preventive or high-risk postpartum populations.

### 3.6 Meta-regression, sensitivity analysis, and publication bias

Key baseline characteristics were generally well balanced across intervention groups, with no clear evidence of substantial imbalance. Univariable network meta-regression showed no statistically significant effect modification by country or region. In contrast, mean age, intervention duration, and intervention frequency were associated with effect estimates in some comparisons, suggesting that these study-level covariates may have functioned as effect modifiers in specific intervention contrasts (**Supplementary Tables S5**–**S8**).

Leave-one-out sensitivity analyses were performed to evaluate the influence of individual studies on the network effect estimates. Overall, the direction of effect remained unchanged across most comparisons after sequential exclusion of single studies, suggesting reasonable robustness of the overall network findings. However, robustness differed across comparisons. For SAE versus PC, the effect estimate consistently favored SAE across all 20 estimable repeat analyses; in 19 of the 20 analyses, the 95% confidence interval did not cross the line of no effect, and only exclusion of Study 13 rendered the result marginally non-significant. This suggests that the advantage of SAE over PC was relatively robust. In contrast, although the direction of effect for MBE versus PC consistently favored MBE, its statistical significance was more sensitive to the exclusion of individual studies, indicating lower stability for this comparison. For LPA versus PC, E+CI versus PC, PFMT versus PC, and AC versus PC, the 95% confidence intervals continued to cross the line of no effect after exclusion of any single study, indicating that the conclusion of no statistically significant difference was relatively stable across these comparisons. Detailed results are presented in **Supplementary Table S4**.

Because the E+CI node represented multicomponent intervention packages rather than exercise-only interventions, its comparative estimates and ranking results could not be attributed to exercise alone. Therefore, due to the potential confounding effects of multi-component interventions, a sensitivity analysis was conducted by excluding the E+CI node. The results showed that both the direction and statistical significance of all intervention effects compared with PC remained unchanged, indicating that the findings were stable and robust. The detailed results are presented in **Supplementary Figure S3**.

For the primary outcome, publication bias and small-study effects were assessed using a comparison-adjusted funnel plot. The plot appeared broadly symmetrical, with no obvious systematic asymmetry or extreme outliers, suggesting a generally low risk of publication bias and small-study effects. The comparison-adjusted funnel plot is presented in **Supplementary Figure S1**. In addition, Egger’s regression test provided no statistical evidence of funnel plot asymmetry for the primary outcome (p = 0.433), further supporting a low likelihood of publication bias and small-study effects, as reported in **Supplementary Table S10**.

### 3.7 Certainty of evidence assessment

The certainty of evidence for the network meta-analysis was assessed using the GRADE framework in conjunction with the CINeMA approach (**Supplementary Table 10**). Overall, the certainty of evidence across intervention comparisons was predominantly low or very low. Comparisons with higher certainty were generally supported by a greater number of direct head-to-head studies and showed more consistent effect directions. In contrast, comparisons rated as low or very low certainty were often characterized by limited sample sizes, wide confidence intervals, sparse network evidence, and greater reliance on indirect evidence.

More specifically, several included studies were rated as having some concerns or high risk in the RoB 2.0 assessment, resulting in downgrading for within-study bias. Although the global inconsistency test did not identify statistically significant inconsistency, some nodes were supported by only a small number of studies, and the distribution of network evidence was uneven, leaving residual concerns regarding indirectness and inconsistency. In addition, several key comparisons were characterized by wide 95% confidence intervals, some of which crossed the line of no effect, indicating important limitations related to imprecision. Taken together, although the findings suggest that MBE and SAE may offer relative advantages for improving postpartum depression scores, the overall certainty of the available evidence remains limited, and these conclusions should be interpreted cautiously. Detailed certainty assessments are presented in **Supplementary Table S9**.

## 4 Discussion

### 4.1 Principal findings

A variety of exercise rehabilitation interventions are currently available for postpartum depression, but direct head-to-head evidence comparing different intervention types remains limited. Accordingly, this study used network meta-analysis to integrate direct and indirect evidence and systematically compare the relative effects of multiple exercise-based interventions. In total, 23 randomized controlled trials involving 2,752 postpartum women were included, generating an evidence network comprising 8 intervention/control nodes.

Taken together, the relative effect estimates, ranking metrics, sensitivity analyses, and certainty-of-evidence assessments suggest that MBE and SAE showed relatively favorable profiles in the current network; however, the nature and strength of the supporting evidence differed between the two interventions. MBE had the highest SUCRA ranking overall, but this ranking was accompanied by limited certainty and less robust statistical evidence in key comparisons. SAE ranked slightly lower but showed clearer statistically significant effects and greater robustness in sensitivity analyses. Subgroup findings further suggest that intervention ranking patterns may differ according to baseline depressive status. Among postpartum women with baseline depression or depressive symptoms above the threshold, MBE and SAE appeared to confer more pronounced relative advantages. By contrast, among women without baseline depression but considered high-risk or included in preventive samples, SAE, E+CI, and PFMT showed relatively favorable ranking profiles.

Importantly, although this study identified several promising exercise-based interventions, the certainty of evidence for most comparisons remained low or very low. The current findings should therefore be viewed as helping to define evidence-based directions rather than as supporting strong or one-size-fits-all clinical recommendations. Accordingly, the principal value of this study lies not in identifying a single “best” intervention but in providing a more nuanced comparative evidence base to inform the selection of exercise rehabilitation strategies for postpartum depression across different clinical settings.

### 4.2 Interpretation of findings

This study found that MBE showed a favorable profile both in the overall ranking and in the subgroup of postpartum women with baseline depression, and this pattern is biologically and behaviorally plausible. MBE primarily includes yoga, mindfulness-based movement, breathing regulation, and other interventions that combine physical activity with elements of psychological regulation. Compared with exercise modalities that mainly emphasize energy expenditure, such interventions may act across multiple domains simultaneously, including emotion regulation, anxiety reduction, sleep improvement, and enhanced body awareness. Previous research suggests that yoga and other mind–body exercises may help alleviate perinatal depressive and anxiety symptoms, with potential mechanisms involving reduced psychological stress, improved sleep, enhanced parasympathetic activity, and better emotional regulation (8, 38). In addition, postpartum anxiety and depressive symptoms have been associated with reduced heart rate variability, further supporting the potential benefits of mind–body exercise from the perspective of autonomic nervous system function (39). For postpartum women with established depressive symptoms, this dual mechanism—combining physical activation with emotional regulation—may be more beneficial than simply increasing activity levels alone.

By contrast, SAE showed clearer statistical advantages in the primary analysis and greater robustness in sensitivity analyses, suggesting that it may be one of the most consistently supported exercise interventions in the current evidence base. Structured aerobic exercise is typically characterized by clearly defined frequency, duration, and implementation procedures, which may facilitate consistency across studies and improve feasibility for implementation in both clinical and community settings. Its potential mechanisms may include behavioral activation, improved sleep and reduced fatigue, enhanced self-efficacy, modulation of neuroendocrine and inflammatory responses, and restoration of daily functioning and activity rhythms through more regularized movement patterns. Recent systematic reviews and meta-analyses have shown that postpartum physical activity can reduce depressive and anxiety symptoms and lower the risk of postpartum depression (8). In addition, the antidepressant effects of physical activity may be partly related to the regulation of brain-derived neurotrophic factor (BDNF) (40). Previous pairwise meta-analyses and network meta-analyses have also generally supported the beneficial effects of aerobic exercise on postpartum depressive symptoms (9, 10). The present study further suggests that, within a relative comparative framework, SAE not only showed a favorable direction of effect but also demonstrated greater stability than some control interventions.

Subgroup findings suggest that the most favorable intervention may differ between therapeutic and preventive populations. Among postpartum women with baseline depression or depressive symptoms above the threshold, MBE had a higher SUCRA ranking, suggesting a potentially favorable direction of effect; however, this ranking does not constitute definitive evidence of superiority. By contrast, among women without baseline depression but considered high-risk, SAE had a higher SUCRA ranking. This finding suggests a potentially favorable role for structured aerobic exercise in preventive settings, but it should be interpreted cautiously because most comparisons were not statistically significant. This distinction indicates that the mechanisms relevant to the treatment and prevention of postpartum depression may not be identical and further highlights the need for more tailored exercise prescriptions according to baseline depressive status in both research and practice.

### 4.3 Implications for clinical practice and research

The present findings suggest that exercise rehabilitation should not be viewed merely as a general lifestyle recommendation in the management of postpartum depression, but should be more explicitly integrated into postpartum mental health care pathways. For postpartum women with depressive symptoms, and in the absence of contraindications to exercise, MBE and SAE may be considered as candidate non-pharmacological options within a shared decision-making framework. In particular, MBE may be especially appealing for women who also experience prominent anxiety, sleep disturbance, emotional tension, or reduced tolerance for higher-intensity activity. By contrast, SAE may offer greater practical advantages in settings where reproducibility, protocol standardization, and scalability are of particular importance.

For women without baseline depression but considered high-risk or included in preventive populations, the present findings suggest that SAE may be considered a potentially suitable option, while E+CI and PFMT may also warrant further investigation. However, the available evidence does not support definitive prioritization of any single intervention. This may be especially relevant in community, primary care, postpartum follow-up, and maternal–child health settings, where structured aerobic exercise offers advantages such as relatively low cost, clear implementation pathways, and ease of integration into routine care, thereby increasing its potential for broader dissemination.

From a research perspective, future studies should move beyond repeatedly asking whether exercise is effective and instead address more clinically informative questions, such as which types of exercise are best suited to specific populations, whether different exercise doses and delivery modes influence treatment effects, and whether combining exercise with other supportive interventions yields synergistic benefits. At the same time, standardizing depression outcome measures, improving the reporting of adverse events, extending follow-up duration, and strengthening head-to-head randomized controlled trial designs will be essential for improving both the usability of the evidence and its clinical interpretability.

### 4.4 Comparison with previous studies

The present findings are broadly consistent with those of previous conventional systematic reviews and meta-analyses, which have generally supported the beneficial effects of exercise interventions on postpartum depressive symptoms (9, 10). However, compared with earlier studies, the present study offers three main contributions.

First, most previous studies have focused primarily on whether exercise is beneficial, rather than directly comparing the relative efficacy of different exercise modalities. Using network meta-analysis, the present study evaluated MBE, SAE, LPA, RT, PFMT, and E+CI within a single comparative framework and further distinguished AC from PC, thereby improving the clinical specificity and interpretability of the comparisons. Second, previous studies have often pooled therapeutic and preventive samples, whereas the present study conducted subgroup analyses according to baseline depressive status. The findings suggest that the more favorable interventions may differ across clinical scenarios, thereby increasing the population specificity of the results. Third, some earlier network meta-analyses have placed substantial emphasis on SUCRA rankings when interpreting results. In contrast, the present study considered relative effect estimates, 95% confidence intervals, sensitivity analyses, and CINeMA/GRADE certainty-of-evidence assessments alongside ranking metrics, thereby avoiding conclusions based solely on rank position.

Some divergence from the existing evidence also merits attention. Previous studies have generally supported the beneficial effects of aerobic exercise on postpartum depressive symptoms (9, 10), and the present study extends this observation by suggesting that SAE not only showed a favorable direction of effect in relative comparisons but also demonstrated greater robustness in key comparisons. At the same time, although MBE had the highest SUCRA ranking in the present analysis, its relative effects were more sensitive to the influence of individual studies in sensitivity analyses, indicating that treatment ranking and result stability do not always coincide. Accordingly, rather than simply interpreting the intervention with the highest SUCRA value as the optimal option, the present findings may be better understood within a layered framework: MBE may be viewed as a potentially promising intervention based on its ranking profile, whereas SAE currently has clearer statistical support and more stable evidence in key comparisons.

### 4.5 Strengths and limitations

This study has several methodological strengths. First, it was conducted in accordance with the PRISMA-NMA statement and prospectively registered, thereby enhancing transparency and reproducibility. Second, a comprehensive search strategy was applied across multiple databases and supplemented by searches of clinical trial registries and citation tracking to reduce the likelihood of missing relevant studies. Third, the analysis was restricted to randomized controlled trials and employed a frequentist random-effects network meta-analysis. Multi-arm trials were appropriately handled, and global consistency assessment, sensitivity analyses, subgroup analyses, and network meta-regression were performed, thereby strengthening the robustness of the findings. Fourth, the intervention nodes were classified in a relatively refined and clinically meaningful manner, with MBE, SAE, LPA, RT, PFMT, and E+CI treated as distinct nodes and active control differentiated from passive control, thereby improving the precision and clinical interpretability of the comparisons. Fifth, RoB 2.0 and CINeMA/GRADE were used to systematically assess risk of bias and certainty of evidence, allowing interpretation of the findings to extend beyond statistical significance alone to a broader consideration of evidential reliability.

Several limitations should also be acknowledged. First, because of the inherent nature of exercise interventions, blinding of participants and intervention providers is often difficult to achieve. Consequently, most included studies were rated as having some concerns in the RoB 2.0 assessment, which may have reduced confidence in the effect estimates. Second, although no significant global inconsistency was identified, some nodes were informed by only a small number of studies, and several comparisons relied heavily on indirect evidence, thereby limiting the certainty of the findings. Third, heterogeneity remained across studies with respect to exercise modality, frequency, duration, implementation setting, and co-interventions. Although these sources of variability were addressed using a random-effects model and network meta-regression, their influence could not be fully eliminated. In particular, the E+CI node should be interpreted with additional caution. This node represented multicomponent intervention packages in which exercise was combined with non-exercise components such as dietary intervention, psychological support, health promotion, or behavioral management. Therefore, the observed effects, SUCRA ranking, and comparative estimates for E+CI cannot be attributed to exercise alone. Although a sensitivity analysis excluding the E+CI node suggested that the main findings were stable, the ranking and comparative results involving E+CI should be regarded as evidence for combined intervention packages rather than for isolated exercise effects. Fourth, the distinction between PC and AC may also involve some judgment, especially when usual care was combined with additional health education or supportive contact. In this study, usual care without additional structured content was classified as PC, whereas usual care combined with structured health education, attention, social support, or planned health-promotion contact was classified as AC. Because AC and PC were prespecified as separate comparator nodes and the number of studies in several control categories was limited, we did not perform an additional sensitivity analysis by reclassifying control conditions. To improve transparency, study-level judgment criteria for each control condition were provided in **Supplementary Table S3**. Fifth, several key comparisons were associated with wide 95% confidence intervals, some of which crossed the line of no effect, indicating important imprecision. Sixth, although the funnel plot and assessment of small-study effects did not indicate clear publication bias, the relatively small sample sizes of some studies mean that publication bias and small-study effects cannot be ruled out entirely. Finally, reporting of long-term follow-up outcomes, adverse events, and adherence remains limited in the existing literature, which constrains conclusions regarding long-term effectiveness and safety.

### 4.6 Future directions

Future research should prioritize head-to-head randomized controlled trials comparing different exercise modalities, particularly direct comparisons between MBE and SAE, to better clarify their relative efficacy. At the same time, there is a need for greater standardization of core depression outcome measures and assessment time points, more consistent reporting of adverse events and adherence, and longer follow-up periods to evaluate the durability of intervention effects. Future studies should also place greater emphasis on potential effect modifiers, including baseline depression severity, postpartum timing, intervention dose, co-interventions, and implementation setting, in order to provide a stronger basis for genuinely individualized postpartum exercise prescriptions.

### 4.7 Conclusion

In summary, SAE currently has the clearest statistically supported comparative evidence and showed relatively robust results in sensitivity analyses. MBE had the highest SUCRA ranking, but its key comparisons with passive and active control were not statistically significant, and the certainty of evidence remained limited. The relative ranking of interventions may vary according to baseline depressive status; however, these subgroup findings should be considered exploratory. Larger, high-quality head-to-head randomized controlled trials are needed before firm conclusions regarding the clinical prioritization of specific exercise modalities can be established.

## Data Availability

The original contributions presented in the study are included in the article/**Supplementary Material**. Further inquiries can be directed to the corresponding author.
