# Supplementary material for: Comparative effectiveness and certainty of exercise-based interventions for postpartum depressive symptoms: a systematic review and network meta-analysis of randomized controlled trials
Source: Front Psychiatry. 2026 Aug 3;17:1889186. doi: 10.3389/fpsyt.2026.1889186 (PMC13478091; doi:10.3389/fpsyt.2026.1889186)
Supplement: Supplementary file 1 [file Supplementaryfile1.docx]

**Supplementary Tables**

**Table S1. Complete database-specific search strategies**

All databases were searched from inception to March 1, 2026. No restrictions were imposed on language, country, or region. Search strategies were adapted to the syntax and indexing system of each database. Controlled vocabulary terms were used where available, including MeSH terms in PubMed and the Cochrane Library and Emtree terms in Embase. Randomized controlled trial filters were applied to bibliographic databases. ClinicalTrials.gov, WHO ICTRP, and PROSPERO were additionally searched using simplified condition- and intervention-related terms to identify completed, ongoing, unpublished, or registered studies.

| **Database/source** | **Search date** | **Field restrictions / controlled vocabulary** | **RCT filter** | **Complete search strategy** |
| --- | --- | --- | --- | --- |
| PubMed | March 1, 2026 | MeSH terms and Title/Abstract fields. No language, country, or region restrictions. | Yes | #1 "Postpartum Period"[Mesh] OR postpartum[Title/Abstract] OR postnatal[Title/Abstract] OR puerperium[Title/Abstract] OR puerperal[Title/Abstract] OR "postpartum women"[Title/Abstract] OR "women, postpartum"[Title/Abstract] #2 "Depression, Postpartum"[Mesh] OR "Depressive Disorder"[Mesh] OR "postpartum depression"[Title/Abstract] OR "postnatal depression"[Title/Abstract] OR "perinatal depression"[Title/Abstract] OR "depressive symptoms"[Title/Abstract] OR "postpartum depressive symptoms"[Title/Abstract] OR "postnatal depressive symptoms"[Title/Abstract] #3 "Exercise"[Mesh] OR "Exercise Therapy"[Mesh] OR "Motor Activity"[Mesh] #4 exercise[Title/Abstract] OR exercises[Title/Abstract] OR "physical activity"[Title/Abstract] OR "exercise therapy"[Title/Abstract] OR "exercise training"[Title/Abstract] OR walking[Title/Abstract] OR "aerobic exercise"[Title/Abstract] OR "resistance training"[Title/Abstract] OR "strength training"[Title/Abstract] OR "pelvic floor muscle training"[Title/Abstract] OR PFMT[Title/Abstract] OR yoga[Title/Abstract] OR "laughter yoga"[Title/Abstract] OR "mind-body exercise"[Title/Abstract] OR mindfulness[Title/Abstract] OR breathing[Title/Abstract] OR "tai chi"[Title/Abstract] #5 randomized controlled trial[Publication Type] OR controlled clinical trial[Publication Type] OR randomized[Title/Abstract] OR randomised[Title/Abstract] OR randomly[Title/Abstract] OR placebo[Title/Abstract] OR trial[Title/Abstract] OR "clinical trial"[Title/Abstract] OR "controlled trial"[Title/Abstract] #6 #1 AND #2 AND (#3 OR #4) AND #5 |
| Embase | March 1, 2026 | Emtree terms and title/abstract/keyword fields. No language, country, or region restrictions. | Yes | #1 'puerperium'/exp OR 'postpartum period'/exp OR postpartum:ti,ab,kw OR postnatal:ti,ab,kw OR puerperium:ti,ab,kw OR puerperal:ti,ab,kw OR 'postpartum women':ti,ab,kw OR 'women postpartum':ti,ab,kw #2 'postnatal depression'/exp OR 'depression'/exp OR 'depressive disorder'/exp OR 'postpartum depression':ti,ab,kw OR 'postnatal depression':ti,ab,kw OR 'perinatal depression':ti,ab,kw OR 'depressive symptoms':ti,ab,kw OR 'postpartum depressive symptoms':ti,ab,kw OR 'postnatal depressive symptoms':ti,ab,kw #3 'exercise'/exp OR 'exercise therapy'/exp OR 'physical activity'/exp #4 exercise:ti,ab,kw OR exercises:ti,ab,kw OR 'physical activity':ti,ab,kw OR 'exercise therapy':ti,ab,kw OR 'exercise training':ti,ab,kw OR walking:ti,ab,kw OR 'aerobic exercise':ti,ab,kw OR 'resistance training':ti,ab,kw OR 'strength training':ti,ab,kw OR 'pelvic floor muscle training':ti,ab,kw OR pfmt:ti,ab,kw OR yoga:ti,ab,kw OR 'laughter yoga':ti,ab,kw OR 'mind body exercise':ti,ab,kw OR mindfulness:ti,ab,kw OR breathing:ti,ab,kw OR 'tai chi':ti,ab,kw #5 'randomized controlled trial'/exp OR 'controlled clinical trial'/exp OR random*:ti,ab,kw OR randomi*:ti,ab,kw OR placebo*:ti,ab,kw OR trial:ti,ab,kw OR 'clinical trial':ti,ab,kw OR 'controlled trial':ti,ab,kw #6 #1 AND #2 AND (#3 OR #4) AND #5 |
| Cochrane Library | March 1, 2026 | MeSH descriptors and title/abstract/keyword fields in trial records. No language, country, or region restrictions. | Yes; trial terms included. Searches were conducted in Cochrane trial records. | #1 MeSH descriptor: [Postpartum Period] explode all trees #2 postpartum OR postnatal OR puerperium OR puerperal OR "postpartum women" OR "women postpartum" #3 #1 OR #2 #4 MeSH descriptor: [Depression, Postpartum] explode all trees #5 MeSH descriptor: [Depressive Disorder] explode all trees #6 "postpartum depression" OR "postnatal depression" OR "perinatal depression" OR "depressive symptoms" OR "postpartum depressive symptoms" OR "postnatal depressive symptoms" #7 #4 OR #5 OR #6 #8 MeSH descriptor: [Exercise] explode all trees #9 MeSH descriptor: [Exercise Therapy] explode all trees #10 exercise OR exercises OR "physical activity" OR "exercise therapy" OR "exercise training" OR walking OR "aerobic exercise" OR "resistance training" OR "strength training" OR "pelvic floor muscle training" OR PFMT OR yoga OR "laughter yoga" OR "mind-body exercise" OR mindfulness OR breathing OR "tai chi" #11 #8 OR #9 OR #10 #12 random* OR randomi* OR placebo* OR trial OR "clinical trial" OR "controlled clinical trial" OR RCT #13 #3 AND #7 AND #11 AND #12 |
| Web of Science Core Collection | March 1, 2026 | Topic field (TS). No controlled vocabulary available. No language, country, or region restrictions. | Yes | #1 TS=(postpartum OR postnatal OR puerperium OR puerperal OR "postpartum women" OR "women postpartum") #2 TS=("postpartum depression" OR "postnatal depression" OR "perinatal depression" OR "depressive symptoms" OR "postpartum depressive symptoms" OR "postnatal depressive symptoms") #3 TS=(exercise OR exercises OR "physical activity" OR "exercise therapy" OR "exercise training" OR walking OR "aerobic exercise" OR "resistance training" OR "strength training" OR "pelvic floor muscle training" OR PFMT OR yoga OR "laughter yoga" OR "mind-body exercise" OR mindfulness OR breathing OR "tai chi") #4 TS=(random* OR randomi* OR randomly OR placebo* OR sham OR trial OR "clinical trial" OR "controlled clinical trial" OR "single blind" OR "double blind" OR "triple blind" OR crossover OR "cross over" OR "parallel group" OR "cluster random*" OR RCT) #5 #1 AND #2 AND #3 AND #4 |
| ClinicalTrials.gov | March 1, 2026 | Condition/disease and other terms fields. No language, country, or region restrictions. | No formal RCT filter; trial registry records were screened for randomized controlled designs. | Condition/disease: postpartum depression OR postnatal depression OR perinatal depression Other terms: exercise OR physical activity OR walking OR aerobic exercise OR resistance training OR pelvic floor muscle training OR yoga OR mindfulness OR mind-body exercise |
| WHO ICTRP | March 1, 2026 | Standard ICTRP search fields. No language, country, or region restrictions. | No formal RCT filter; registry records were screened for randomized controlled designs. | postpartum depression AND exercise postnatal depression AND exercise perinatal depression AND exercise postpartum depression AND physical activity postpartum depression AND yoga postpartum depression AND walking postpartum depression AND pelvic floor muscle training |
| PROSPERO | March 1, 2026 | Title/abstract/keyword search fields. No language, country, or region restrictions. | Not applied; records were screened for relevant systematic review registrations. | postpartum depression AND exercise postnatal depression AND exercise perinatal depression AND physical activity postpartum depression AND network meta-analysis postpartum depression AND systematic review |

Table S2. Definitions of intervention and control nodes and study-level rationale for node assignment

| **Node** | **Definition** | **Examples / classification criteria** |
| --- | --- | --- |
| LPA | Lifestyle physical activity promotion interventions primarily aimed at increasing daily physical activity, usually delivered in a flexible and non-strictly supervised manner. | Telephone support, social support, web-based platforms, behavioral promotion strategies |
| SAE | Structured aerobic exercise delivered according to a clear exercise prescription. | Pram walking, aerobic gymnastics, supervised or home-based aerobic exercise with predefined frequency, duration, or session structure |
| MBE | Interventions combining physical activity with psychological, breathing, or attentional regulation components. | Yoga, laughter yoga, mindfulness-based movement, breathing-regulation exercise, or VR-enhanced mindfulness plus yoga |
| RT | Interventions centered on muscle strengthening or resistance training. | Progressive resistance training, strength training, or supervised muscle-strengthening exercise |
| PFMT | Interventions primarily focused on pelvic floor muscle training or pelvic floor rehabilitation. | Repeated pelvic floor muscle contractions, pelvic floor rehabilitation, or supervised pelvic floor muscle training |
| E+CI | Multicomponent interventions in which exercise is combined with diet, psychological support, health promotion, or other components, such that the independent effect of exercise cannot be isolated. | Dietary intervention, psychological support, behavioral management |
| PC | Passive control | Usual care, wait-list control, no intervention, or standard postpartum management without structured exercise, health education, social support, or other active intervention components. |
| AC | Active control | Non-primary exercise comparator conditions involving active contact, attention, or non-exercise support, including health education, attention control, social support, general health promotion, or non-targeted exercise interventions that were not designed as the main therapeutic exercise intervention. |

Part B. Study-level rationale for intervention and control node assignment

| **Study** | **Assigned intervention node** | **Control node** | **Rationale for node assignment** |
| --- | --- | --- | --- |
| Keller, 2014 | LPA | AC | The intervention primarily aimed to promote daily physical activity and walking through social support and behavioral strategies; therefore, it was classified as LPA. The comparator provided attention/routine health information without equivalent exercise promotion and was classified as AC. |
| Forsyth, 2017 | SAE | PC | The intervention involved pram-walking, group exercise, and home exercise as an adjunctive exercise treatment; therefore, it was classified as SAE. The comparator was usual care and was classified as PC. |
| Da Costa, 2009 | SAE | PC | The intervention was a supervised individualized home-based exercise program with a clear exercise structure; therefore, it was classified as SAE. The comparator was usual care and was classified as PC. |
| Daley, 2008 | LPA | PC | The intervention used exercise consultations and telephone support to encourage gradual physical activity, mainly walking and pram-pushing, without a strict supervised exercise prescription; therefore, it was classified as LPA. |
| Glazener, 2001 | PFMT | PC | The intervention centered on pelvic floor muscle training and postpartum pelvic floor rehabilitation; therefore, it was classified as PFMT. |
| Armstrong, 2004 | SAE | AC | The intervention was a pram-walking exercise program and was classified as SAE. The comparator was a social support group without structured exercise and was classified as AC. |
| Heh, 2008 | SAE | PC | The intervention included hospital-based exercise support plus home exercise with defined frequency; therefore, it was classified as SAE. |
| Buttner, 2015 | MBE | PC | The intervention was yoga, which combines physical movement with breathing and psychological/attentional regulation; therefore, it was classified as MBE. |
| Teychenne, 2021 | LPA | PC | The intervention was a home-based physical activity promotion program using equipment, a smartphone app, an online forum, and logbooks; therefore, it was classified as LPA rather than structured aerobic exercise. |
| Daley, 2015 | LPA | PC | The intervention used consultations and telephone support to facilitate physical activity behavior change; therefore, it was classified as LPA. |
| Lewis, 2014 | LPA | AC | The intervention was a telephone-delivered home-based physical activity intervention and was classified as LPA. The wellness/support contact comparator was classified as AC. |
| Lewis, 2021 | LPA | AC/PC | The exercise arm was telephone-based physical activity promotion and was classified as LPA. The wellness/support arm was classified as AC, and usual care was classified as PC. |
| Özkan, 2020 | SAE | PC | The intervention was a structured exercise program with a defined duration; therefore, it was classified as SAE. |
| Norman, 2010 | SAE | AC | The intervention combined exercise with health education, while the comparator received education only. Because the core differential component was structured exercise, it was classified as SAE and the education-only comparator as AC. |
| Surkan, 2012 | E+CI | PC | The intervention combined physical activity promotion with diet, self-efficacy, social support, home visits, and motivational counseling, making the independent effect of exercise difficult to isolate; therefore, it was classified as E+CI. |
| Yang, 2018 | SAE | PC | The intervention was home-based aerobic gymnastic exercise with defined frequency and session duration; therefore, it was classified as SAE. |
| LeCheminant, 2014 | RT | AC | The intervention was resistance training and was classified as RT. The flexibility training comparator was classified as AC. |
| Armstrong, 2003 | LPA | PC | The intervention combined exercise and social support, with physical activity promotion as the core component; therefore, it was classified as LPA. The comparator was non-intervention control and was classified as PC. |
| Haruna, 2013 | SAE | PC | The intervention consisted of scheduled ball-exercise classes and was classified as SAE. |
| Thiruppathi, 2014 | SAE | AC | The intervention involved structured physical activity plus health care education, while the comparator received health care education only. Because the core differential component was structured physical activity, it was classified as SAE and the comparator as AC. |
| Ko, 2008 | SAE | PC | The intervention was a low-intensity postpartum exercise program delivered in a postpartum care center with a minimum number of sessions; therefore, it was classified as SAE. |
| Koca, 2025 | MBE | PC | The intervention was laughter yoga, which combines bodily movement, breathing/laughter practice, and emotional regulation; therefore, it was classified as MBE. |
| Liu, 2025 | MBE | AC | The intervention was VR-enhanced mindfulness plus yoga, combining physical movement with mindfulness and psychological regulation; therefore, it was classified as MBE. The traditional in-person session comparator was classified as AC. |

Table S3. Detailed intervention characteristics and depression outcome measures of included studies

| **First author and year** | **Detailed intervention in the intervention group** | **Detailed intervention in the control group** | **Control node and judgment basis** | **Depression outcome measure** |
| --- | --- | --- | --- | --- |
| Keller, 2014 | Social support–facilitated moderate-intensity physical activity program centered on walking/physical activity promotion, combined with weight management and behavioral support. | Attention control / routine health information without equivalent exercise promotion. | AC; attention control/routine health information without equivalent exercise promotion, representing structured non-exercise contact. | Depression score |
| Forsyth, 2017 | Pram-walking, group exercise, and self-initiated home exercise as an adjunctive treatment for postpartum depression. | Usual care. | PC; usual care without additional structured trial-delivered education, attention, social support, or health-promotion contact. | EPDS |
| Da Costa, 2009 | 12-week individualized home-based exercise program supervised by professionals. | Usual care. | PC; usual care without additional structured trial-delivered education, attention, social support, or health-promotion contact. | HAM-D |
| Daley, 2008 | Two one-to-one exercise consultations plus telephone support to encourage the gradual establishment of regular moderate-intensity activity, mainly including walking and pram-pushing. | Usual care. | PC; usual care without additional structured trial-delivered education, attention, social support, or health-promotion contact. | EPDS |
| Glazener, 2001 | Nurse assessment plus pelvic floor muscle training, with additional bladder training when necessary. | Standard management. | PC; standard management without additional structured trial-delivered education, attention, social support, or health-promotion contact. | HAD depression |
| Armstrong, 2004 | Pram-walking program. | Social support group involving non-structured supportive activities. | AC; social support group involving structured supportive contact without structured exercise. | EPDS |
| Heh, 2008 | Exercise support program: 1 hour per week in hospital plus home exercise twice weekly. | Standard care. | PC; standard care without additional structured trial-delivered education, attention, social support, or health-promotion contact. | EPDS |
| Buttner, 2015 | Yoga intervention, 16 sessions over 8 weeks. | Wait-list control. | PC; wait-list control representing a delayed-treatment/no active comparator condition. | HDRS |
| Teychenne, 2021 | Home-based multicomponent physical activity promotion program including free equipment, smartphone app, online forum, and logbook. | Usual routine. | PC; usual routine care without additional structured trial-delivered education, attention, social support, or health-promotion contact. | EPDS |
| Daley, 2015 | Facilitated exercise intervention involving two face-to-face consultations and two telephone support sessions. | Usual care only. | PC; usual care only without additional structured trial-delivered education, attention, social support, or health-promotion contact. | EPDS |
| Lewis, 2014 | Telephone-delivered home-based physical activity intervention lasting 6 months. | Wellness/support contact control discussing stress, sleep, nutrition, and related topics, but not focused on exercise promotion. | AC; wellness/support contact discussing stress, sleep, nutrition, and related topics, representing structured non-exercise supportive contact. | EPDS |
| Lewis, 2021 | Telephone-based exercise intervention. | Wellness/support intervention plus usual care; active control arm in a three-arm trial. | AC; wellness/support intervention plus usual care, representing structured non-exercise supportive contact in a three-arm trial. | EPDS |
| Özkan, 2020 | 4-week structured exercise program. | Standard care. | PC; standard care without additional structured trial-delivered education, attention, social support, or health-promotion contact. | EPDS |
| Norman, 2010 | Mother and Baby program: exercise plus health care education. | Education only. | AC; education-only comparator representing structured non-exercise educational contact. | EPDS |
| Surkan, 2012 | JFY health promotion program including home visits and motivational telephone counseling to promote diet, physical activity, self-efficacy, and social support. | Usual WIC care. | PC; usual WIC care treated as routine care without additional trial-delivered structured exercise or non-exercise contact. | CES-D |
| Yang, 2018 | Home-based aerobic gymnastic exercise at least 3 times per week, 15 minutes per session. | Routine care / control. | PC; routine care/control without additional structured trial-delivered education, attention, social support, or health-promotion contact. | EPDS |
| LeCheminant, 2014 | Resistance training twice weekly for 18 weeks. | Flexibility training (active control). | AC; flexibility training used as an active non-primary exercise comparator to control for contact and activity exposure. | CES-D |
| Armstrong, 2003 | Multicomponent program combining exercise and social support. | Non-intervention control. | PC; non-intervention control without additional structured trial-delivered education, attention, social support, or health-promotion contact. | EPDS |
| Haruna, 2013 | Ball-exercise classes once weekly for 90 minutes over 4 weeks. | Control / usual care. | PC; control/usual care without additional structured trial-delivered education, attention, social support, or health-promotion contact. | EPDS |
| Thiruppathi, 2014 | Structured physical activity plus health care education. | Health care education only. | AC; health care education-only comparator representing structured non-exercise educational contact. | EPDS |
| Ko, 2008 | Low-intensity postpartum exercise program in a postpartum care center, with at least 6 completed sessions. | Traditional postpartum care without a physical activity component. | PC; traditional postpartum care without a physical activity component or additional structured trial-delivered education/support. | CES-D |
| Koca, 2025 | Laughter Yoga, delivered in 6 sessions. | Control group without laughter yoga. | PC; control group without laughter yoga and without additional structured active comparator components. | EPDS |
| Liu, 2025 | VR-enhanced mindfulness plus yoga, 3 times per week, 60 minutes per session. | Traditional in-person sessions / active control; part of a three-arm trial. | AC; traditional in-person sessions/active control, representing structured contact in a three-arm trial. | EPDS |

Table S4. Leave-one-out sensitivity analysis for depression score outcome

| **First author, year** | **comparison** | **eff** | **lci** | **uci** | **connected** |
| --- | --- | --- | --- | --- | --- |
| Keller, 2014 | E+CI vs AC | 0.247027 | -0.720502 | 1.214557 | 1 |
| Keller, 2014 | LPA vs AC | -0.197976 | -2.205518 | 1.809566 | 1 |
| Keller, 2014 | MBE vs AC | -0.354525 | -1.22282 | 0.513769 | 1 |
| Keller, 2014 | PC vs AC | -1.056224 | -2.292766 | 0.180318 | 1 |
| Keller, 2014 | PFMT vs AC | -0.1092 | -2.115599 | 1.897199 | 1 |
| Keller, 2014 | RT vs AC | -0.408119 | -2.710065 | 1.893827 | 1 |
| Keller, 2014 | SAE vs AC | -0.82761 | -1.544987 | -0.110232 | 1 |
| Forsyth, 2017 | E+CI vs AC | 0.129921 | -0.79002 | 1.049863 | 1 |
| Forsyth, 2017 | LPA vs AC | -0.197976 | -2.185734 | 1.789782 | 1 |
| Forsyth, 2017 | MBE vs AC | -0.312266 | -1.146817 | 0.522284 | 1 |
| Forsyth, 2017 | PC vs AC | -1.095298 | -2.317055 | 0.126458 | 1 |
| Forsyth, 2017 | PFMT vs AC | -0.1092 | -2.095804 | 1.877404 | 1 |
| Forsyth, 2017 | RT vs AC | -0.525224 | -2.790195 | 1.739747 | 1 |
| Forsyth, 2017 | SAE vs AC | -0.931001 | -1.679169 | -0.182834 | 1 |
| Da Costa, 2009 | E+CI vs AC | 0.149062 | -0.784107 | 1.082232 | 1 |
| Da Costa, 2009 | LPA vs AC | -0.197975 | -2.21364 | 1.81769 | 1 |
| Da Costa, 2009 | MBE vs AC | -0.305759 | -1.15134 | 0.539823 | 1 |
| Da Costa, 2009 | PC vs AC | -1.088983 | -2.326986 | 0.14902 | 1 |
| Da Costa, 2009 | PFMT vs AC | -0.109199 | -2.123726 | 1.905327 | 1 |
| Da Costa, 2009 | RT vs AC | -0.506083 | -2.800931 | 1.788766 | 1 |
| Da Costa, 2009 | SAE vs AC | -0.894685 | -1.658097 | -0.131273 | 1 |
| Daley, 2008 | E+CI vs AC | 0.163642 | -0.776836 | 1.104119 | 1 |
| Daley, 2008 | LPA vs AC | -0.197976 | -2.215117 | 1.819165 | 1 |
| Daley, 2008 | MBE vs AC | -0.312177 | -1.232628 | 0.608275 | 1 |
| Daley, 2008 | PC vs AC | -1.084116 | -2.323535 | 0.155303 | 1 |
| Daley, 2008 | PFMT vs AC | -0.1092 | -2.125204 | 1.906803 | 1 |
| Daley, 2008 | RT vs AC | -0.491504 | -2.790629 | 1.80762 | 1 |
| Daley, 2008 | SAE vs AC | -0.851454 | -1.56896 | -0.133949 | 1 |
| Glazener, 2001 | E+CI vs AC | 0.165554 | -0.728548 | 1.059657 | 1 |
| Glazener, 2001 | LPA vs AC | -0.197976 | -2.141097 | 1.745145 | 1 |
| Glazener, 2001 | MBE vs AC | -0.297365 | -1.113414 | 0.518684 | 1 |
| Glazener, 2001 | PC vs AC | -1.083253 | -2.27865 | 0.112143 | 1 |
| Glazener, 2001 | PFMT vs AC | -0.489591 | -2.704938 | 1.725755 | 1 |
| Glazener, 2001 | RT vs AC | -0.846503 | -1.537988 | -0.155017 | 1 |
| Armstrong, 2004 | E+CI vs AC | 0.159748 | -0.811865 | 1.13136 | 1 |
| Armstrong, 2004 | LPA vs AC | -0.197976 | -2.208043 | 1.812091 | 1 |
| Armstrong, 2004 | MBE vs AC | -0.301536 | -1.151444 | 0.548373 | 1 |
| Armstrong, 2004 | PC vs AC | -1.085397 | -2.32366 | 0.152866 | 1 |
| Armstrong, 2004 | PFMT vs AC | -0.1092 | -2.118126 | 1.899726 | 1 |
| Armstrong, 2004 | RT vs AC | -0.495398 | -2.801264 | 1.810468 | 1 |
| Armstrong, 2004 | SAE vs AC | -0.844884 | -1.571267 | -0.118501 | 1 |
| Heh, 2008 | E+CI vs AC | 0.158317 | -0.776366 | 1.093 | 1 |
| Heh, 2008 | LPA vs AC | -0.197976 | -2.217546 | 1.821595 | 1 |
| Heh, 2008 | MBE vs AC | -0.302345 | -1.149423 | 0.544733 | 1 |
| Heh, 2008 | PC vs AC | -1.085902 | -2.326151 | 0.154348 | 1 |
| Heh, 2008 | PFMT vs AC | -0.1092 | -2.127635 | 1.909235 | 1 |
| Heh, 2008 | RT vs AC | -0.496828 | -2.795723 | 1.802066 | 1 |
| Heh, 2008 | SAE vs AC | -0.875514 | -1.639391 | -0.111636 | 1 |
| Buttner, 2015 | E+CI vs AC | 0.102973 | -0.819585 | 1.025532 | 1 |
| Buttner, 2015 | LPA vs AC | -0.197976 | -2.171238 | 1.775286 | 1 |
| Buttner, 2015 | MBE vs AC | -0.322127 | -1.152357 | 0.508104 | 1 |
| Buttner, 2015 | PC vs AC | -1.428972 | -2.940192 | 0.082249 | 1 |
| Buttner, 2015 | PFMT vs AC | -0.1092 | -2.081299 | 1.862899 | 1 |
| Buttner, 2015 | RT vs AC | -0.552172 | -2.805502 | 1.701158 | 1 |
| Buttner, 2015 | SAE vs AC | -0.865792 | -1.568861 | -0.162724 | 1 |
| Teychenne, 2021 | E+CI vs AC | 0.150298 | -0.790961 | 1.091556 | 1 |
| Teychenne, 2021 | LPA vs AC | -0.197976 | -2.214719 | 1.818767 | 1 |
| Teychenne, 2021 | MBE vs AC | -0.34141 | -1.266151 | 0.583331 | 1 |
| Teychenne, 2021 | PC vs AC | -1.088574 | -2.327843 | 0.150696 | 1 |
| Teychenne, 2021 | PFMT vs AC | -0.1092 | -2.124805 | 1.906406 | 1 |
| Teychenne, 2021 | RT vs AC | -0.504848 | -2.803943 | 1.794247 | 1 |
| Teychenne, 2021 | SAE vs AC | -0.855161 | -1.572626 | -0.137697 | 1 |
| Daley, 2015 | E+CI vs AC | 0.181342 | -0.762194 | 1.124877 | 1 |
| Daley, 2015 | LPA vs AC | -0.197976 | -2.21904 | 1.823089 | 1 |
| Daley, 2015 | MBE vs AC | -0.273863 | -1.202874 | 0.655148 | 1 |
| Daley, 2015 | PC vs AC | -1.078213 | -2.320019 | 0.163594 | 1 |
| Daley, 2015 | PFMT vs AC | -0.1092 | -2.129129 | 1.910729 | 1 |
| Daley, 2015 | RT vs AC | -0.473804 | -2.777622 | 1.830014 | 1 |
| Daley, 2015 | SAE vs AC | -0.84673 | -1.56568 | -0.12778 | 1 |
| Lewis, 2014 | E+CI vs AC | 0.158677 | -0.815114 | 1.132468 | 1 |
| Lewis, 2014 | LPA vs AC | -0.197976 | -2.219874 | 1.823922 | 1 |
| Lewis, 2014 | MBE vs AC | -0.289092 | -1.163005 | 0.58482 | 1 |
| Lewis, 2014 | PC vs AC | -1.085788 | -2.330645 | 0.159069 | 1 |
| Lewis, 2014 | PFMT vs AC | -0.1092 | -2.129963 | 1.911563 | 1 |
| Lewis, 2014 | RT vs AC | -0.496468 | -2.81357 | 1.820633 | 1 |
| Lewis, 2014 | SAE vs AC | -0.853114 | -1.575282 | -0.130945 | 1 |
| Lewis, 2021 | E+CI vs AC | 0.241273 | -0.839927 | 1.322473 | 1 |
| Lewis, 2021 | LPA vs AC | -0.197975 | -2.292464 | 1.896514 | 1 |
| Lewis, 2021 | MBE vs AC | -0.366349 | -1.329434 | 0.596737 | 1 |
| Lewis, 2021 | PC vs AC | -1.05839 | -2.352174 | 0.235394 | 1 |
| Lewis, 2021 | PFMT vs AC | -0.1092 | -2.202593 | 1.984194 | 1 |
| Lewis, 2021 | RT vs AC | -0.413873 | -2.840494 | 2.012748 | 1 |
| Lewis, 2021 | SAE vs AC | -0.833901 | -1.588478 | -0.079324 | 1 |
| Özkan, 2020 | E+CI vs AC | 0.247427 | -0.599315 | 1.094168 | 1 |
| Özkan, 2020 | LPA vs AC | -0.197976 | -2.011721 | 1.615769 | 1 |
| Özkan, 2020 | MBE vs AC | -0.261664 | -1.028656 | 0.505328 | 1 |
| Özkan, 2020 | PC vs AC | -1.055429 | -2.176928 | 0.066071 | 1 |
| Özkan, 2020 | PFMT vs AC | -0.1092 | -1.92168 | 1.70328 | 1 |
| Özkan, 2020 | RT vs AC | -0.40772 | -2.49081 | 1.675371 | 1 |
| Özkan, 2020 | SAE vs AC | -0.642907 | -1.332527 | 0.046713 | 1 |
| Norman, 2010 | E+CI vs AC | 0.2986 | -0.675346 | 1.272547 | 1 |
| Norman, 2010 | LPA vs AC | -0.197976 | -2.186385 | 1.790433 | 1 |
| Norman, 2010 | MBE vs AC | -0.248013 | -1.091498 | 0.595472 | 1 |
| Norman, 2010 | PC vs AC | -1.038932 | -2.265696 | 0.187832 | 1 |
| Norman, 2010 | PFMT vs AC | -0.1092 | -2.096455 | 1.878055 | 1 |
| Norman, 2010 | RT vs AC | -0.356546 | -2.64455 | 1.931458 | 1 |
| Norman, 2010 | SAE vs AC | -0.906147 | -1.628336 | -0.183958 | 1 |
| Surkan, 2012 | E+CI vs AC | 0.165554 | -0.728548 | 1.059657 | 1 |
| Surkan, 2012 | LPA vs AC | -0.297365 | -1.113414 | 0.518684 | 1 |
| Surkan, 2012 | MBE vs AC | -1.083253 | -2.27865 | 0.112143 | 1 |
| Surkan, 2012 | PC vs AC | -0.1092 | -2.05114 | 1.83274 | 1 |
| Surkan, 2012 | PFMT vs AC | -0.489591 | -2.704938 | 1.725755 | 1 |
| Surkan, 2012 | RT vs AC | -0.846503 | -1.537988 | -0.155017 | 1 |
| Yang, 2018 | E+CI vs AC | 0.10452 | -0.80057 | 1.009609 | 1 |
| Yang, 2018 | LPA vs AC | -0.197976 | -2.147939 | 1.751987 | 1 |
| Yang, 2018 | MBE vs AC | -0.320866 | -1.140877 | 0.499145 | 1 |
| Yang, 2018 | PC vs AC | -1.103673 | -2.303697 | 0.096351 | 1 |
| Yang, 2018 | PFMT vs AC | -0.1092 | -2.057986 | 1.839586 | 1 |
| Yang, 2018 | RT vs AC | -0.550626 | -2.776421 | 1.675169 | 1 |
| Yang, 2018 | SAE vs AC | -0.979013 | -1.719994 | -0.238032 | 1 |
| LeCheminant, 2014 | E+CI vs AC | 0.165554 | -0.72855 | 1.059658 | 1 |
| LeCheminant, 2014 | LPA vs AC | -0.197976 | -2.141098 | 1.745147 | 1 |
| LeCheminant, 2014 | MBE vs AC | -0.297365 | -1.113415 | 0.518684 | 1 |
| LeCheminant, 2014 | PC vs AC | -1.083254 | -2.278651 | 0.112144 | 1 |
| LeCheminant, 2014 | PFMT vs AC | -0.1092 | -2.051142 | 1.832742 | 1 |
| LeCheminant, 2014 | RT vs AC | -0.846503 | -1.537989 | -0.155017 | 1 |
| Armstrong, 2003 | E+CI vs AC | 0.26047 | -0.62985 | 1.150789 | 1 |
| Armstrong, 2003 | LPA vs AC | -0.197976 | -2.101965 | 1.706014 | 1 |
| Armstrong, 2003 | MBE vs AC | -0.083955 | -0.946514 | 0.778604 | 1 |
| Armstrong, 2003 | PC vs AC | -1.0514 | -2.225295 | 0.122494 | 1 |
| Armstrong, 2003 | PFMT vs AC | -0.1092 | -2.011985 | 1.793585 | 1 |
| Armstrong, 2003 | RT vs AC | -0.394677 | -2.574234 | 1.784881 | 1 |
| Armstrong, 2003 | SAE vs AC | -0.817666 | -1.497294 | -0.138039 | 1 |
| Haruna, 2013 | E+CI vs AC | 0.121496 | -0.797436 | 1.040428 | 1 |
| Haruna, 2013 | LPA vs AC | -0.197976 | -2.180462 | 1.78451 | 1 |
| Haruna, 2013 | MBE vs AC | -0.315328 | -1.147987 | 0.517331 | 1 |
| Haruna, 2013 | PC vs AC | -1.098099 | -2.31691 | 0.120713 | 1 |
| Haruna, 2013 | PFMT vs AC | -0.1092 | -2.090529 | 1.872129 | 1 |
| Haruna, 2013 | RT vs AC | -0.533649 | -2.793585 | 1.726286 | 1 |
| Haruna, 2013 | SAE vs AC | -0.948182 | -1.700102 | -0.196261 | 1 |
| Thiruppathi, 2014 | E+CI vs AC | -0.265597 | -0.950723 | 0.419529 | 1 |
| Thiruppathi, 2014 | LPA vs AC | -0.197976 | -1.586875 | 1.190923 | 1 |
| Thiruppathi, 2014 | MBE vs AC | -0.443621 | -1.051795 | 0.164553 | 1 |
| Thiruppathi, 2014 | PC vs AC | -1.224883 | -2.106296 | -0.34347 | 1 |
| Thiruppathi, 2014 | PFMT vs AC | -0.1092 | -1.496447 | 1.278046 | 1 |
| Thiruppathi, 2014 | RT vs AC | -0.920742 | -2.573338 | 0.731854 | 1 |
| Thiruppathi, 2014 | SAE vs AC | -0.63809 | -1.154698 | -0.121482 | 1 |
| Ko, 2008 | E+CI vs AC | 0.136945 | -0.790586 | 1.064477 | 1 |
| Ko, 2008 | LPA vs AC | -0.197976 | -2.200997 | 1.805045 | 1 |
| Ko, 2008 | MBE vs AC | -0.31002 | -1.150648 | 0.530608 | 1 |
| Ko, 2008 | PC vs AC | -1.092996 | -2.323666 | 0.137674 | 1 |
| Ko, 2008 | PFMT vs AC | -0.1092 | -2.111076 | 1.892676 | 1 |
| Ko, 2008 | RT vs AC | -0.5182 | -2.799654 | 1.763253 | 1 |
| Ko, 2008 | SAE vs AC | -0.918529 | -1.676596 | -0.160461 | 1 |
| Koca, 2025 | E+CI vs AC | 0.315726 | -0.477385 | 1.108837 | 1 |
| Koca, 2025 | LPA vs AC | -0.197976 | -1.869598 | 1.473647 | 1 |
| Koca, 2025 | MBE vs AC | -0.229452 | -0.942667 | 0.483763 | 1 |
| Koca, 2025 | PC vs AC | -0.174565 | -1.467877 | 1.118747 | 1 |
| Koca, 2025 | PFMT vs AC | -0.1092 | -1.77945 | 1.56105 | 1 |
| Koca, 2025 | RT vs AC | -0.339421 | -2.277455 | 1.598613 | 1 |
| Koca, 2025 | SAE vs AC | -0.785593 | -1.389374 | -0.181811 | 1 |
| Liu, 2025 | E+CI vs AC | 0.399825 | -0.537042 | 1.336693 | 1 |
| Liu, 2025 | LPA vs AC | -0.197976 | -2.089916 | 1.693965 | 1 |
| Liu, 2025 | MBE vs AC | -0.206123 | -1.012881 | 0.600635 | 1 |
| Liu, 2025 | PC vs AC | -1.604846 | -2.988378 | -0.221314 | 1 |
| Liu, 2025 | PFMT vs AC | -0.1092 | -1.999928 | 1.781528 | 1 |
| Liu, 2025 | RT vs AC | -0.255322 | -2.443879 | 1.933236 | 1 |
| Liu, 2025 | SAE vs AC | -0.778248 | -1.459237 | -0.09726 | 1 |

Table S5. Network meta-regression analysis of age on depression score outcome

| **Intervention comparison** | **Covariate** | **Coefficient** | **Std.err.** | **z** | **P>z** | **Lower 95% CI** | **Upper 95% CI** |
| --- | --- | --- | --- | --- | --- | --- | --- |
| E+CI vs AC | cons | -1.51 | 1.11 | -1 | 0.18 | -3.69 | 0.68 |
| LPA vs AC | age | 0.47 | 0.23 | 2 | 0.04 | 0.03 | 0.92 |
| LPA vs AC | cons | -14.54 | 6.81 | -2 | 0.03 | -27.90 | -1.19 |
| MBE vs AC | age | 2.16 | 0.87 | 2 | 0.01 | 0.45 | 3.87 |
| MBE vs AC | cons | -66.71 | 26.55 | -3 | 0.01 | -118.74 | -14.68 |
| PC vs AC | age | 0.40 | 0.21 | 2 | 0.06 | -0.01 | 0.82 |
| PC vs AC | cons | -11.94 | 6.34 | -2 | 0.06 | -24.37 | 0.48 |
| PFMT vs AC | cons | -0.21 | 0.86 | 0 | 0.80 | -1.89 | 1.47 |
| RT vs AC | cons | -0.66 | 0.82 | -1 | 0.43 | -2.27 | 0.96 |
| SAE vs AC | age | 0.54 | 0.20 | 3 | 0.01 | 0.15 | 0.93 |
| SAE vs AC | cons | -16.99 | 5.86 | -3 | 0.00 | -28.47 | -5.50 |

Table S6. Network meta-regression analysis of country/region on depression score outcome

| **Intervention comparison** | **Covariate** | **Coefficient** | **Std.err.** | **z** | **P>z** | **Lower 95% CI** | **Upper 95% CI** |
| --- | --- | --- | --- | --- | --- | --- | --- |
| E+CI vs AC | cons | -0.3602492 | 1.249061 | -0.29 | 0.773 | -2.808365 | 2.087866 |
| LPA vs AC | country_num | -0.312616 | 0.3511161 | -0.89 | 0.373 | -1.000791 | 0.3755589 |
| LPA vs AC | cons | 0.109253 | 0.784548 | 0.14 | 0.889 | -1.428433 | 1.646939 |
| MBE vs AC | country_num | -0.0243523 | 0.1837518 | -0.13 | 0.895 | -0.3844992 | 0.3357946 |
| MBE vs AC | cons | -1.223286 | 1.383567 | -0.88 | 0.377 | -3.935026 | 1.488455 |
| PC vs AC | country_num | -0.0616825 | 0.1686598 | -0.37 | 0.715 | -0.3922495 | 0.2688846 |
| PC vs AC | cons | -0.1005933 | 0.8439828 | -0.12 | 0.905 | -1.754769 | 1.553583 |
| PFMT vs AC | cons | -0.5182027 | 1.164984 | -0.44 | 0.656 | -2.80153 | 1.765125 |
| RT vs AC | cons | -0.6556301 | 1.061598 | -0.62 | 0.537 | -2.736324 | 1.425064 |
| SAE vs AC | country_num | -0.3449615 | 0.1980054 | -1.74 | 0.081 | -0.733045 | 0.0431219 |
| SAE vs AC | cons | 0.6759262 | 1.112929 | 0.61 | 0.544 | -1.505375 | 2.857228 |

Table S7. Network meta-regression analysis of intervention duration on depression score outcome

| **Intervention comparison** | **Covariate** | **Coefficient** | **Std.err.** | **z** | **P>z** | **Lower 95% CI** | **Upper 95% CI** |
| --- | --- | --- | --- | --- | --- | --- | --- |
| E+CI vs AC | cons | 1.004252 | 2.041929 | 0.49 | 0.623 | -2.997855 | 5.006359 |
| LPA vs AC | dur_wk | 0.0285372 | 0.0274615 | 1.04 | 0.299 | -0.0252864 | 0.0823608 |
| LPA vs AC | cons | -1.228593 | 0.8552196 | -1.44 | 0.151 | -2.904793 | 0.4476065 |
| MBE vs AC | dur_wk | 1.436746 | 0.5592883 | 2.57 | 0.01 | 0.3405609 | 2.532931 |
| MBE vs AC | cons | -12.18495 | 4.250943 | -2.87 | 0.004 | -20.51665 | -3.853259 |
| PC vs AC | dur_wk | 0.0436335 | 0.0476478 | 0.92 | 0.36 | -0.0497544 | 0.1370215 |
| PC vs AC | cons | -1.066713 | 0.7947203 | -1.34 | 0.18 | -2.624337 | 0.4909098 |
| PFMT vs AC | cons | 0.3948929 | 1.409679 | 0.28 | 0.779 | -2.368028 | 3.157813 |
| RT vs AC | cons | -0.6556302 | 0.886474 | -0.74 | 0.46 | -2.393087 | 1.081827 |
| SAE vs AC | dur_wk | 0.1683433 | 0.0843512 | 2 | 0.046 | 0.003018 | 0.3336685 |
| SAE vs AC | cons | -2.794721 | 0.9134998 | -3.06 | 0.002 | -4.585148 | -1.004295 |

Table S8. Network meta-regression analysis of intervention frequency on depression score outcome

| **Intervention comparison** | **Covariate** | **Coefficient** | **Std.err.** | **z** | **P>z** | **Lower 95% CI** | **Upper 95% CI** |
| --- | --- | --- | --- | --- | --- | --- | --- |
| E+CI vs AC | cons | -0.5627529 | 1.06186 | -0.53 | 0.596 | -2.643961 | 1.518455 |
| LPA vs AC | freq_wk | 0.2832051 | 0.3039945 | 0.93 | 0.352 | -0.3126131 | 0.8790232 |
| LPA vs AC | cons | -1.764114 | 1.214916 | -1.45 | 0.146 | -4.145306 | 0.6170775 |
| MBE vs AC | freq_wk | 1.84357 | 0.7402242 | 2.49 | 0.013 | 0.3927575 | 3.294383 |
| MBE vs AC | cons | -5.523305 | 1.863283 | -2.96 | 0.003 | -9.175273 | -1.871337 |
| PC vs AC | freq_wk | 0.193603 | 0.306941 | 0.63 | 0.528 | -0.4079904 | 0.7951963 |
| PC vs AC | cons | -1.332794 | 1.116266 | -1.19 | 0.232 | -3.520635 | 0.8550463 |
| PFMT vs AC | cons | -0.0867732 | 1.458601 | -0.06 | 0.953 | -2.945579 | 2.772032 |
| RT vs AC | cons | -0.6556301 | 0.8732043 | -0.75 | 0.453 | -2.367079 | 1.055819 |
| SAE vs AC | freq_wk | -0.5294344 | 0.3467817 | -1.53 | 0.127 | -1.209114 | 0.1502452 |
| SAE vs AC | cons | -0.0957574 | 1.01591 | -0.09 | 0.925 | -2.086905 | 1.89539 |

Table S9. CINeMA/GRADE certainty assessment for network estimates

| **Comparison** | **Number of studies** | **Within-study bias** | **Reporting bias** | **Indirectness** | **Imprecision** | **Heterogeneity** | **Incoherence** | **Confidence** | **Reason**  **(s) for downgrading** |
| --- | --- | --- | --- | --- | --- | --- | --- | --- | --- |
| AC:LPA | 3 | Some concerns | Low risk | No concerns | Some concerns | Some concerns | No concerns | Very low | Within-study bias; Imprecision; Heterogeneity |
| AC:MBE | 1 | Some concerns | Low risk | No concerns | No concerns | Some concerns | No concerns | Low | Within-study bias; Heterogeneity |
| AC:PC | 1 | Some concerns | Low risk | No concerns | Some concerns | No concerns | No concerns | Low | Within-study bias; Imprecision |
| AC:RT | 1 | Some concerns | Low risk | No concerns | Some concerns | No concerns | No concerns | Low | Within-study bias; Imprecision |
| AC:SAE | 3 | Some concerns | Low risk | No concerns | No concerns | Some concerns | No concerns | Low | Within-study bias; Heterogeneity |
| E+CI:PC | 1 | Some concerns | Low risk | No concerns | Some concerns | No concerns | No concerns | Low | Within-study bias; Imprecision |
| LPA:PC | 5 | No concerns | Low risk | No concerns | Some concerns | Some concerns | No concerns | Low | Imprecision; Heterogeneity |
| MBE:PC | 2 | Some concerns | Low risk | No concerns | No concerns | Some concerns | No concerns | Low | Within-study bias; Heterogeneity |
| PC:PFMT | 1 | Some concerns | Low risk | No concerns | Some concerns | No concerns | No concerns | Low | Within-study bias; Imprecision |
| PC:SAE | 7 | Some concerns | Low risk | No concerns | No concerns | Some concerns | Some concerns | Very low | Within-study bias; Heterogeneity; Incoherence |
| AC:E+CI | 0 | Some concerns | Low risk | No concerns | Some concerns | No concerns | No concerns | Low | Within-study bias; Imprecision |
| AC:PFMT | 0 | Some concerns | Low risk | No concerns | Some concerns | No concerns | No concerns | Low | Within-study bias; Imprecision |
| E+CI:LPA | 0 | Some concerns | Low risk | No concerns | Some concerns | No concerns | No concerns | Low | Within-study bias; Imprecision |
| E+CI:MBE | 0 | Some concerns | Low risk | No concerns | Some concerns | No concerns | No concerns | Low | Within-study bias; Imprecision |
| E+CI:PFMT | 0 | Some concerns | Low risk | No concerns | Some concerns | No concerns | No concerns | Low | Within-study bias; Imprecision |
| E+CI:RT | 0 | Some concerns | Low risk | No concerns | Some concerns | No concerns | No concerns | Low | Within-study bias; Imprecision |
| E+CI:SAE | 0 | Some concerns | Low risk | No concerns | Some concerns | No concerns | No concerns | Low | Within-study bias; Imprecision |
| LPA:MBE | 0 | Some concerns | Low risk | No concerns | Some concerns | Some concerns | No concerns | Very low | Within-study bias; Imprecision; Heterogeneity |
| LPA:PFMT | 0 | Some concerns | Low risk | No concerns | Some concerns | No concerns | No concerns | Low | Within-study bias; Imprecision |
| LPA:RT | 0 | Some concerns | Low risk | No concerns | Some concerns | No concerns | No concerns | Low | Within-study bias; Imprecision |
| LPA:SAE | 0 | Some concerns | Low risk | No concerns | Some concerns | Some concerns | No concerns | Very low | Within-study bias; Imprecision; Heterogeneity |
| MBE:PFMT | 0 | Some concerns | Low risk | No concerns | Some concerns | No concerns | No concerns | Low | Within-study bias; Imprecision |
| MBE:RT | 0 | Some concerns | Low risk | No concerns | Some concerns | No concerns | No concerns | Low | Within-study bias; Imprecision |
| MBE:SAE | 0 | Some concerns | Low risk | No concerns | Some concerns | No concerns | No concerns | Low | Within-study bias; Imprecision |
| PC:RT | 0 | Some concerns | Low risk | No concerns | Some concerns | No concerns | No concerns | Low | Within-study bias; Imprecision |
| PFMT:RT | 0 | Some concerns | Low risk | No concerns | Some concerns | No concerns | No concerns | Low | Within-study bias; Imprecision |
| PFMT:SAE | 0 | Some concerns | Low risk | No concerns | Some concerns | No concerns | No concerns | Low | Within-study bias; Imprecision |
| RT:SAE | 0 | Some concerns | Low risk | No concerns | Some concerns | No concerns | No concerns | Low | Within-study bias; Imprecision |

Table S10. Regression test for small-study effects for depression score

| Item | Result |
| --- | --- |
| Null hypothesis | H0: beta1 = 0; no small-study effects |
| beta1 | -0.97 |
| SE of beta1 | 1.232 |
| z | -0.78 |
| P value | 0.433 |
| Interpretation | No statistically significant small-study effects |

Figure S1: Comparison-adjusted funnel plot for depression score

Figure S2: League table of subgroup analysis for depression score by baseline depressive status

**Figure S3. League table from the sensitivity analysis excluding the E+CI node**
